# Histone modification profiles are predictive for tissue/cell-type specific expression of both protein-coding and microRNA genes

**DOI:** 10.1186/1471-2105-12-155

**Published:** 2011-05-14

**Authors:** Zhihua Zhang, Michael Q Zhang

**Affiliations:** 1 Department of Molecular Cell Biology, Center for Systems Biology, University of Texas at Dallas, 800 W Campbell Road, Richardson, TX 75080, USA

## Abstract

**Background:**

Gene expression is regulated at both the DNA sequence level and through modification of chromatin. However, the effect of chromatin on tissue/cell-type specific gene regulation (TCSR) is largely unknown. In this paper, we present a method to elucidate the relationship between histone modification/variation (HMV) and TCSR.

**Results:**

A classifier for differentiating CD4+ T cell-specific genes from housekeeping genes using HMV data was built. We found HMV in both promoter and gene body regions to be predictive of genes which are targets of TCSR. For example, the histone modification types H3K4me3 and H3K27ac were identified as the most predictive for CpG-related promoters, whereas H3K4me3 and H3K79me3 were the most predictive for nonCpG-related promoters. However, genes targeted by TCSR can be predicted using other type of HMVs as well. Such redundancy implies that multiple type of underlying regulatory elements, such as enhancers or intragenic alternative promoters, which can regulate gene expression in a tissue/cell-type specific fashion, may be marked by the HMVs. Finally, we show that the predictive power of HMV for TCSR is not limited to protein-coding genes in CD4+ T cells, as we successfully predicted TCSR targeted genes in muscle cells, as well as microRNA genes with expression specific to CD4+ T cells, by the same classifier which was trained on HMV data of protein-coding genes in CD4+ T cells.

**Conclusion:**

We have begun to understand the HMV patterns that guide gene expression in both tissue/cell-type specific and ubiquitous manner.

## Background

The development of a human body from a single fertilized egg is a spatially and temporally regulated complex process. The genes that are responsible for general cellular function are expressed in all cell-types and tissues. However, in many tissue/cell-types, specialized functions require or exclude the expression of certain genes. The mechanism of this tissue/cell-type specific regulation (TCSR) is rather intriguing. It is worth noting that such diverse expression patterns are achieved through one genome shared largely by all cells. Gene transcription is regulated in multiple layers, e.g. transcription factor binding through DNA nucleotide features, DNA methylations, and chromatin modifications. TCSR may involve combinations of these regulations in all layers (for review [1-3]).

Thanks to next generation sequencing technology, our understanding of human TCSR has accelerated in recent years. At the base layer of DNA features, the association between DNA regulatory elements, such as TATA box and CpG islands in the promoter regions, and tissue-specific regulation has been investigated experimentally [1] and computationally [4]; Tissue-specific regulatory transcription factor binding sites in the promoter regions have been well studied in muscle [5] and liver [6], and binding sites were also detected in multiple tissues using generic transcription factor binding site prediction tools [7-9]. Cell-type specific enhancers have been experimentally explored in several cell types as well [10]. High-throughput Cap Analysis of Gene Expression (CAGE) data showed that alternative transcription start sites (TSS) exist in the mammalian genome with more prevalence than previously thought [11], and, moreover, distributions of TSS have also been associated with TCSR [12]. Recently, genome-wide mapping of Histone Modifications and Variants (HMVs) in CD4+ T cells [13,14], as well as other cell types [15], opened up an opportunity to model gene expression levels from the perspective of post-translational modification of histones [16]. For example, Pekowska *et al. *clustered genes by their H3K4me2 profile at the promoter regions in CD4+ T cells. They found that a cluster was enriched in CD4+ T cell specific genes [17]. However, a comprehensive picture on how posttranslational modifications of histones contribute to TCSR is still not clear.

Therefore, in this work, we addressed three major questions 1) which HMVs carry sufficient information to allow TCSR target gene prediction, 2) whether TCSR is the same as gene expression activity regulation, and 3) whether the predictive relationship between HMV and TCSR target genes is universal for entire Pol II transcriptome. To properly address these questions, we developed a quantitative model to link the HMVs and TCSR target genes using CoreBoost, and applied it to recently published genome-wide mapped HMVs in CD4+ T cells [13,14]. CoreBoost is a previously developed boosting classifier [18,19] that can select informative features from an ensemble of weak classifiers. We first show that HMV profiles in both proximal promoters and gene bodies are predictive for CD4+ T cell specificity. The most predictive HMV types have been identified for CpG- and nonCpG-related genes in promoters and gene bodies. The evidences have shown that the underlying enhancers and intragenic alternative promoters marked by the HMV patterns were associated with tissue/cell-type specific gene expression. Second, we demonstrated that TCSR is different from the regulation of gene expression activity. Finally, the model, which was trained on HMV data of protein-coding genes in CD4+ T cells, successfully predicted muscle cell specific genes and CD4+ T cell specific microRNA genes.

## Results and Discussion

### Definition of CD4+ T cell specific regulated genes

We chose CD4+ T cells as the model, taking advantage of the widespread availability of genome-wide HMV data for this cell type [13,14]. CD4+ T cell specific expressed genes (denoted as CD4SE) and housekeeping genes (denoted as HK) were collected as positive and negative datasets. We identified CD4SE genes according to their expression profiles among human tissues and other information. Altogether, 454 and 630 genes were collected in CD4SE, and HK sets, respectively (see Methods and Materials).

Genes in the CD4SE set were not expressed in most tissue/cell-types other than blood cell types. We plotted the expression distribution of genes in CD4SE, HK and randomly selected genes among all tissues in the GNF symAtlas dataset [20] as shown in Additional file 1. CD4SE genes were only expressed in a small number of blood cell types (CD14, CD19, CD33, CD4, CD56, CD8, X721 B/T cells, and whole blood), as expected, since this result agrees with the high expression correlation between blood cells [15,16]. On the other hand, the HK genes and randomly selected genes were expressed in various tissue/cell-types studied. Quantitatively, both the overall entropy and categorical entropy in CD4+ T cells are significantly smaller in CD4SE genes than in HK genes [4] (the average overall entropies for CD4SE and HK genes are 4.8 and 6.26 as in the GNF symAtlas dataset [4,20], respectively, P < 2.2e-16; the average categorical entropies for CD4SE and HK genes are 8.95 and 12.35 as in the GNF symAtlas dataset respectively, P < 2.2e-16).

### The predictive HMVs for CD4+ T cell specific regulation

Previous studies suggested that CpG- and nonCpG-related promoters have different regulatory characteristics [21-26], and have a contrasting distribution of HMVs [19]. Following the same strategy used previously for CoreBoost [18], CoreBoost_HM [19], and a third work [16], we analyzed CpG- and nonCpG-related genes separately. There were 40 HMV types in the CD4+ T cell dataset [13,14], many of which were correlated with each other [13]. We first performed a principal component analysis (PCA) and grouped the HMVs into two sets. For convenience, we refer to them as Set I and Set II. Set I contained the HMVs that have the highest contributions in the first 4 principal components (which captured 90% of variance, see Additional file 2). Set II contained the remaining HMVs. There were 25 and 15 HMVs in Set I and Set II, respectively. We trained CoreBoost to distinguish between CD4SE genes and HK genes. Because there were more genes in the HK set (630) than in the CD4SE set (454), we randomly sampled about 454 HK genes and combined them with CD4SE to form a total set. The performance of the CoreBoosts was evaluated based on sensitivity, positive predictive value (PPV) [27] and F-score [28]. Five-fold cross-validation was performed to limit over-fitting. To further eliminate any potential bias introduced by sampling fluctuation, we repeated the whole process 100 times.

**Figure 1:**
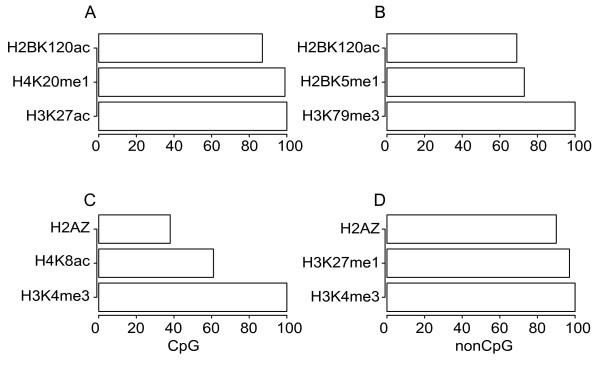
**Top selected HMV features by the TCSR model in proximal promoters**. The *x*-axis shows the number of times in which an HMV feature has been selected as the top predictive feature in 100 replicates. A, B) The HMV features selected from Set I for CpG and nonCpG genes, respectively; C,D) features selected from Set II.

**Figure 2:**
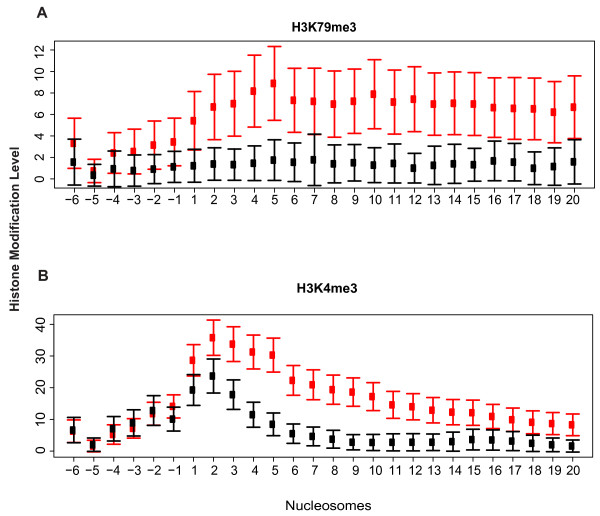
**The distribution of HMV levels in proximal promoter regions**. The dots and bars give the average and standard deviation of HMV levels in each nucleosome, respectively. Data for CD4SE genes are shown in red, and data for HK genes are shown in black. The *x*-axis shows the index of the nucleosomes. The nucleosomes upstream of the TSS are assigned as minus (-) and the nucleosomes downstream of the TSS are assigned as plus (+). A) H3K79me3 and B) H3K4me3 are the most predictive HMV types selected for nonCpG-related genes from Set I and Set II, respectively (see Additional file 3).

We focused first on the features in proximal promoters. The classifiers trained with HMV in the proximal promoters significantly differentiate CD4SE genes from HK genes (Table 1). In both Set I and Set II, CoreBoost performed much better in nonCpG-related genes than in CpG-related genes. This is because CpG-related genes are more likely to be associated with housekeeping genes [4]. A few HMV types have been highlighted as the most predictive features in our 100 replicates (Figure 1 and Additional file 3 and 4), and we noticed that many of those selected features are located downstream of the TSS in both Set I and Set II (Additional file 5). We then plotted the distribution of the selected HMVs among promoter regions (Figure 2). We see that the major difference of the HMV levels between the CD4SE and HK promoters is found downstream of the annotated TSS, indicating that HMV patterns in gene bodies may also be predictive of TCSR targets.

**Table 1:** The performance of CoreBoost based on features in CpG- or nonCpG-related proximal promoter and gene body region

HMV groups			Sensitivity	PPV	F-Score
	Promoter	CpG	0.579 ± 0.016	0.764 ± 0.028	0.658 ± 0.006
		
**Set I**		non-CpG	0.889 ± 0.032	0.771 ± 0.021	0.825 ± 0.007
	
	Body	CpG	0.594 ± 0.013	0.789 ± 0.021	0.678 ± 0.007
		
		non-CpG	0.876 ± 0.015	0.811 ± 0.013	0.842 ± 0.006

	Promoter	CpG	0.523 ± 0.016	0.717 ± 0.021	0.605 ± 0.010
		
**Set II**		non-CpG	0.908 ± 0.033	0.790 ± 0.021	0.844 ± 0.007
	
	Body	CpG	0.588 ± 0.015	0.736 ± 0.020	0.653 ± 0.008
		
		non-CpG	0.892 ± 0.014	0.821 ± 0.012	0.854 ± 0.006

Averages and errors are given as the mean and standard deviation from 100 replicates. All of the CoreBoost performance statistics have a p-value < 1e-5.

To investigate this possibility, we designed a new HMV feature table containing the following information: the average and sum of each HMV level for the first exon and the first intron; the average and sum of each HMV level for the whole gene body; and the sum of each HMV level in the first twenty nucleosomes positioned after the first exon. The first exon and the first intron were chosen because previous studies had shown the first exon and/or intron can play important roles in gene regulation [29], especially in tissue-specific regulation [30,31]. Using this newly designed feature table, we repeated the CoreBoost training and analysis. The "body" entries in Table 1 summarize the performance of the new CoreBoost classifier for 100 replicates. We found that the classifiers have similar performance, irrespective of whether the HMV features in promoter or in gene bodies were used for training, and both performed significantly better than classifiers trained by control regions (Table 1). For CpG-related genes, the features sums of H3K27ac, sums of H3K79me3 and sums of H3K4me3 levels in the entire gene bodies contributed most to the prediction of CD4+ T cell specificity (see Additional file 5). For nonCpG-related gene, the features sums of H4K20me3 and sums of H3K14ac levels in the entire gene bodies contribute most to the prediction (see Additional file 5). Based on this line of evidence, we conclude that HMV profiles in gene bodies encode information about TCSR, much like those in promoters.

### TCSR is different from gene expression activity regulation at the HMV level

We have shown above that TCSR target genes can be predicted by HMV profiles in both promoters and gene body regions. The immediate question that follows is how much gene expression level *per se *may determine TCSR. It might be argued our TCSR model achieves high predictive power is because CD4SE genes are highly expressed in CD4+ T cell and therefore could be easily predicted by any gene expression level prediction model. We now argue that this is not the case.

The predictive power of our TCSR model does not stem from the high expression level of CD4SE genes. First, if we define highly expressed genes as those genes whose expression levels are at least one standard deviation higher than average levels in a given cell type, then CD4SE genes are by no means highly expressed genes, even though they are higher than expression levels of HK genes (rank sum test P = 0.01). Second, our model does not simply predict highly expressed genes as CD4+ T cell specific. For example, of the 159 CpG-related genes that were predicted as CD4+ T cell specific in at least half of 100 replicates, only 26 genes were actually highly expressed in CD4+ T cell (P = 0.005). Moreover, the predicted highly expressed genes by the model proposed by Karlic *et al. *are expressed in broad tissues, but our model predicted CD4+ T cell specific genes expressed only in limited blood cell types akin to CD4SE genes (Additional file 6). Therefore, it is not surprising that our predictions, in comparison, have significantly smaller overall entropies (average overall entropies are 5.7 and 4.3, respectively, rank sum test P < 2.2e-16) and categorical entropies (5.8 and 4.5, respectively, rank sum test P < 2.2e-16). The same observation can be made even if one removes the intersection of the two predictions (overall entropies are 5.7 and 4.3 respectively, P < 2.2e-16; and categorical entropies are 5.9 and 4.6 respectively, P < 2.2e-16).

The difference was also indicated by the distinct HMV types selected as predictive features between our TCSR model and the gene expression activity model proposed by Karlic *et al*. That is, while Karlic *et al. *identified HMV type pairs H4K20me1/H3K27ac and H3K4me3/H3K79me1 as the most powerful predictive features for CpG and nonCpG-related promoters respectively [16], our model identified different HMV type pairs for TCSR (Figure 1, Additional file 3 and 4). To explain, first, we noticed that the second most predictive features selected by CoreBoost for CpG promoters in Set I was H4K20me1 (Figure 1A), which was also one of the two most predictive features selected by Karlic *et al *for gene expression activity prediction. To ensure that the reason why we did not choose H4K20me1 as the most predictive feature was not because of the separation of features into two initial input sets, we retrained our model with the initial input features including all HMVs in the final selection of either models (H4K20me1, H3K27ac, H3K4me3, and H4K8ac for CpG-related promoters, and H3K79me3, H3K79me1, K3K4me3, H2BK5me1, and H3K27me1 for nonCpG-related promoters, see Table 2). Indeed we still found that H3K4me3/H3K27ac and H3K4me3/H3K79me3 remain to be selected by our CoreBoost model as the best predictors of TCSR for CpG- and nonCpG-related promoters, respectively. Second, it is known that some HMVs are highly correlated, for example H3K27ac and H2BK5ac are highly correlated in both CD4SE and highly expressed genes (median values of *r *are 0.91 and 0.78, respectively), but H3K23ac and H4K20me1 are not highly correlated (median values of *r *are 0.1 and 0.09, respectively). Therefore, if the HMV types selected by the two models are truly different, we should expect at least one pairing of HMV types from either model to be poorly correlated in their promoter regions. We compared the Pearson correlation coefficient of the HMV profiles in each case for both CD4SE and highly expressed gene promoters (Table 2). For the HMV type pairs selected by our TCSR model, there is always an HMV type that has low correlation with both of the HMVs selected by the model of Karlic *et al. *This is more so in nonCpG-related promoters than CpG promoters. As we discussed above this is probably related to the fact that CpG-related genes are, in general, largely housekeeping genes. Therefore, the most predictive HMV types which were chosen for TCSR prediction by CoreBoost and the HMV types which have been chosen for gene expression activities by the model of Karlic *et al *were true dissimilar.

**Table 2:** The correlations between predictive HMV profiles in nonCpG-related and CpG-related promoters

			CD4SE		High	
			
			H3K4me3	H3K79me1	H3K4me3	H3K79me1
non-CpG	Set I	H3K79me3	0.346	0.344	0.190	0.420
		H2BK5me1	0.021	0.295	-0.072	0.378
	Set II	H3K4me3	1.000	0.161	1.000	0.127
		H3K27me1	0.006	0.231	0.014	0.355
CpG			H4K20me1	H3K27ac	H4K20me1	H3K27ac
	Set I	H4K20me1	1.000	0.032	1.000	-0.009
		H3K27ac	0.032	1.000	-0.009	1.000
	Set II	H3K4me3	0.121	0.688	-0.013	0.799
		H4K8ac	-0.031	0.512	-0.121	0.748

Two of the most frequently selected HMV types by our CoreBoost model in Set I and Set II are represented in rows for each subset. The two HMV types selected by the model of Karlic *et al *are represented in columns. The numbers are the median values of Pearson's correlation coefficient of HMV profiles in the promoter of CD4SE genes or promoter of highly expressed genes.

### What makes HMV predictive for tissue/cell-types specific regulation?

**Figure 3:**
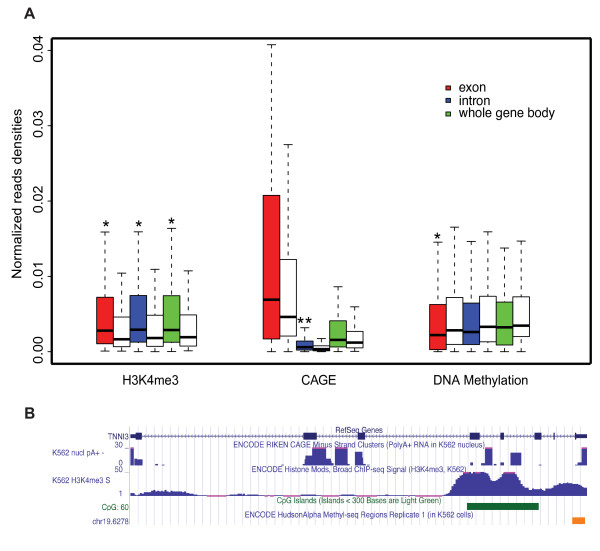
**Alternative promoter activities in the gene body**. A) H3K4me3, CAGE tag and DNA Methylation levels were compared between K562 specific genes and housekeeping genes in the K562 cell line. All data were controlled by region lengths. H3K4me3 and CAGE tag levels have been further controlled by gene expression levels. DNA methylation levels have been further controlled by CpG island lengths. Red, blue, and green boxes represent data in exonic, intronic, and whole gene body regions for K562 specific genes respectively. Blank boxes represent data in housekeeping genes for the same type of regions as the box next to its left. The Wilcoxon rank sum test P-values between the K562 specific genes and housekeeping genes are shown by the '*' (P < 0.05) and '**' (P < 0.0001); B) Detailed data of H3K4me3, CAGE, and DNA methylation density for K562 specific gene *TNNI3*.

We noticed that H3K4me3 had been chosen as the most predictive HMV marks in both the promoter and gene body region for both CpG- and nonCpG-related genes by our TCSR model. H3K4me3 is a well-documented HMV signal that marks the promoter [13,14,25,32-34]. This fact let us to investigate the roles of intragenic alternative promoters for TCSR. To infer the potential promoter activities in gene bodies, we further looked at the capped analysis of gene expression (CAGE) experimental data and DNA methylation data in the gene bodies. Because it is well known that 1) CAGE data directly indicates the transcription initiation site [11], and 2) DNA methylation in promoters suppresses gene expression [35], we used the two datasets as positive and negative controls, respectively. If the alternative promoters in the gene body contribute to TCSR, we should expect to observe relatively higher H3K4me3 levels and CAGE tags in the gene body of tissue/cell specific genes than those of housekeeping genes. On the other hand, a relatively higher DNA methylation level should be observed in gene bodies of housekeeping genes. In the ENCODE project [36], the Broad Institute mapped HMVs for the K562 cell line [24], the RIKEN Institute did CAGE experiments on the same cell line [37], and the DNA methylation level has also been measured in the cell line via Methyl-seq technology by Brunner and colleagues [38]. We identified the K562 specific expressed genes in a manner similar to that used for CD4+ T cells, and all three datasets were compared between K562 specific genes and HK genes in the gene body (Figure 3A). As expected, intragenic H3K4me3 levels are significantly higher in K562 specific genes than in HK genes (P < 0.05). Furthermore, intronic CAGE levels are higher in K562 specific genes than in HK genes (P < 0.001), while exonic DNA methylation levels are generally lower in K562 specific genes than in HK genes (P < 0.05). The association between DNA methylation and promoter is weaker and has longer physical distance in DNA sequences than is CAGE, because the mechanisms by which DNA methylation to suppresses gene expression are circuitous in nature, e.g., by recruitment of methyl-CpG-binding domain proteins (MBD), which, in turn, recruit histone modifying and chromatin-remodeling complexes to the site to change the histone status (for review see [39]). Nevertheless, the data we showed here indicated that the alternative promoters in gene body could associate with TCSR. For example, troponin I type 3 (cardiac), *TNNI3*, is a gene specifically expressed in heart (z-score of categorical entropy > 13) and Leukemia cell lines (the z-score > 2.8 for K562). We saw strong CAGE peaks in the intronic regions. There are CAGE tag peaks found in the third and the fourth introns which have been further marked by high H3K4me3 levels. An unmethylated CpG island covered the region from the third exon to the fifth exon. This corroborating data strongly suggests that specific expression of *TNNI3 *in K562 could be regulated via alternative promoters located in the third and the fourth introns (Figure 3B). Interestingly, Alika and colleagues recently reported that alternative promoters in the gene body of *SHANK3 *regulate human brain specific expression of the gene [40]. The alternative promoters in *SHANK3*'s gene body were marked by high level of H3K4me3, as well as CAGE tags and unmethylated CpG islands.

There are several other possible associations between TCSR and HMV patterns. The HMV patterns could be markers in the nucleosomes indicating enhancers in the nearby DNA sequence. The binding of a transcription regulatory factor at an enhancer has long been suggested as one of the most important mechanisms of tissue/cell-type regulation [10,15,34]. H3K4me1 is most frequently associated with enhancers [10,13]. We compared H3K4me1 profiles in the gene body with the profile of other HMV types (see Additional file 7). For the 15 HMVs which most correlated with H3K4me1, 13 of them (87%, hypergeometric test P = 9.3E-9) were selected as the top predictive features by resampling at least once in the 100 replicates. In addition, there are other HMVs types associated with the enhancers. For example, H2A.Z, H3K27ac, monomethylated H3K4, H3K9, and H3K27 were all found to be strongly associated with enhancers [13-15,32,41,42]. Also, six HMVs (H3K4me1, H3K4me2, H3K4me3, H3K9me1, H3K18ac, and H2A.Z) were detected at more than a fifth of potential enhancers [13]. All of these HMVs were selected as predictive HMVs at least once by resampling (see Additional file 5), indicating the possibility of the underlying enhancer activity in the regions.

Another possibility is that tissue/cell-type specific expression could be regulated after transcription initiation and/or in the pause and elongation stages. Recent studies implied that the majority of genes are transcriptionally initiated and paused [43-45]. H3K79me2, a characteristic marker of RNAPII elongation, is only found downstream of TSS in the human genome [46]. In our data, H3K79me2 is a most frequently selected predictive HMV among the 100 replicates from Set I (see Additional file 4). In nearly all the cases (except for the Set I HMVs in the nonCpG related promoters), as shown in Table 2, we noticed an HMV highly correlated with H3K4me3 (in nonCpG related genes) and H3K27ac (in CpG-related genes), respectively. H3K4me3 and H3K27ac are well-known gene activity markers [13,14]. The other HMVs are much less correlated with either of the HMVs selected by the gene activity model Karlic *et al *(except for the Set II HMVs in the CpG related promoters). Given this observation, we propose that the HMV profile of H3K27ac and H3K4me3, together with other correlated HMV types, may provide a basal layer of information for gene transcriptional regulation in CpG- and nonCpG-related genes, respectively. And as additional signals, the remaining HMV marks may be "modulated" on top of the basal signals so that the tissue/cell-type specificities of gene expression can be achieved. This modulation process could be manifested either by guiding the binding of transcription factors at enhancer regions or by directing the pause or elongation of transcription, as discussed above.

### The HMV profile marks skeletal muscle myoblasts specific genes

We next asked whether the predictive HMV model trained by the CD4+ T cell data could also be used to predict in other cell types. We collected HMV data for normal human skeletal muscle myoblasts (HSMM) from the ENCODE project [36], in the Broad Institute Chip-seq dataset [24]. A total of 416 HSMM specific expressed genes were identified by the same method as used for CD4+ T cells. There were eight HMV types (H3K4me1, H3K4me2, H3K4me3, H3K9ac, H3K27ac, H3K27me3, H3K36me3, and H4K20me1) available in the ENCODE dataset. CoreBoost classifiers were retrained based on these eight HMV types in the CD4+ T cell data. By applying these newly trained classifiers to the HSMM input data, the classifiers successfully predicted HSMM specific genes with similar sensitivities as before with the CD4+ T cell input data (Table 3). However, the specificities (PPV) of the new classifiers were lower than before for CpG related genes (Table 1), possibly because CpG related genes are more likely to be housekeeping genes. Nevertheless, the performance of these newly trained classifiers are significant better than the controls, which were also retrained based on the eight HMV types in the CD4+ T cell data in the control regions and applied to HSMM dataset (Table 3). Therefore, the model we trained by the CD4 T cell data was not specific to the CD4 T cells, and it can be applied to other cell types as well.

**Table 3:** The performance of CoreBoost classifiers

			Sensitivity	PPV	F-Score
HSMM	Promoter	CpG	0.929 ± 0.098	0.467 ± 0.012	0.619 ± 0.033
		non-CpG	0.764 ± 0.214	0.617 ± 0.043	0.662 ± 0.123
	Body	CpG	0.919 ± 0.135	0.473 ± 0.022	0.618 ± 0.043
		non-CpG	0.860 ± 0.183	0.608 ± 0.025	0.700 ± 0.093
miRNA	Promoter	Set I	0.417 ± 0.037	0.441 ± 0.036	0.426 ± 0.011
		Set II	0.365 ± 0.073†	0.521 ± 0.085	0.422 ± 0.079†
	Body	Set I	0.255 ± 0.080	0.220 ± 0.068†	0.234 ± 0.068†
		Set II	0.125 ± 0.057*	0.099 ± 0.069*	0.102 ± 0.056*

In the "HSMM" rows, the classifiers were trained based on eight HMV features in CD4+ T cells, in the proximal promoter and gene body region, respectively. In the "miRNA" rows, the classifiers were trained on full Set I or Set II HMV features. Averages and errors are given as the mean and standard deviation from 100 replicates. The significances of comparison between the performance of CoreBoost trained on features in those regions and control regions are indicated by symbols next to each number. No symbol indicates p-value < 1e-5; * indicates p-value < 1e-2 and > = 1e-5; † indicates p-value > 1e-2.

### Prediction of CD4+ T cell specific regulation of microRNA genes

MicroRNAs (miRNA) are a class of short RNA molecules which are generated from intergenic or intronic transcripts called pri-miRNAs (for review see [47,48]). Similar to mRNA, pri-miRNAs also have a 5' cap structure and a 3' ployA tail [49]. The majority of pri-miRNAs are believed to be transcribed by Pol II [50], with a few exceptions [51]. Nevertheless, most pri-miRNAs share a transcription mechanism similar to protein-coding genes.

To test whether the association between TCSR and HMV patterns we found in protein-coding gene is similar for miRNAs genes, we trained our CoreBoost classifiers using the HMV profiles of protein-coding genes and applied them to miRNA genes. We evaluated our prediction with a recently published miRNA expression atlas [52] in which 13 and 50 CpG-related miRNAs clusters were identified as CD4+ T cell specific and housekeeping, respectively. The performance of the classifiers trained in promoter and gene body was significantly better than the performance of classifiers trained in control regions (Table 3), although they were not as good as the performance for predicting protein-coding genes (Table 1). The relatively lower performance of the classifiers on miRNA most likely results from the fact that we do not have sufficient knowledge about the miRNA gene structures, e.g., the TSS, the full length of pre-miRNA transcript, or the existences or the lengths of first exon/introns. The promoter regions of miRNA genes used for this prediction were obtained by recent computational predictions [19]. However, because of the shortage of high-quality training data, miRNA promoter prediction is a difficult problem, and the resolution and the accuracy of the predictions are relatively lower [19,23]. On the other hand, our classifiers were trained on the HMV profiles in individual hypothetical nucleosomes related to a well-defined TSS. Thus, the low resolution of promoter prediction has a significant effect on the nucleosomes assignment (as 500-bp resolution could end up with a difference of about 3 nucleosomes). This effect lowers the expectation of the predictive power of our HMV promoter trained classifiers. Nevertheless, even without full knowledge, our model was still be able to correctly predict about 40% of CD4+ T cell specific miRNAs, and this prediction was significantly better than the control. This result suggested that miRNA genes may share a similar association between HMV patterns and TCSR with protein-coding genes.

### Predictive information is redundantly distributed among HMVs

In this work, we identified H3K4me3, H3K79me3, and H3K27ac as the most predictive marks in the promoter regions (Figure 1). However, these three HMV marks are by no means the only predictive ones. For example, H3K79me2 has also been selected as the most predictive HMV marks in nonCpG-related gene bodies (see Additional file 4). Therefore, we can reasonably argue that the predictive power for detecting TCSR targeted gene is redundantly encoded among HMVs. One clue indicating the existence of such redundancy was the success of applying our model to HSMM cell input data. Instead of using the full model, we trained our CoreBoost classifiers with the eight HMV types which were available in the ENCODE dataset. Although neither H3K79me2 nor H3K79me3 were available in the ENCODE dataset, the classifiers still managed to make significant predictions with similar performance as those trained with the full HMV set (Table 3).

To further exclude the possibility that this high performance could not be attributed to the existence of one or several dominating HMV marks, we performed the training and testing once more with a subset of HMV type set, in which all three of the most predictive HMV types H3K4me3, H3K79me3, and H3K27ac were removed. We also excluded H3K4me2 from the training data because this HMV type has recently been suggested as a unique mark for CD4+ T cell specificity [17]. Interestingly, the classifiers also achieved similar significant predictive power as the classifiers trained by the full HMV profile (Table 3 and Additional file 8). With the possible exception of H3K9me1, Pekowska *et al. *did not find any other HMV marks than H3K4me2 that could make the same enrichment of CD4+ T cell specific genes [17]. This is probably because clustering did not fully reveal the profound relationship between HMV profile and TCSR. To explore this possibility, we revisited the cluster (cluster 1) in which they observed enrichment of CD4+ T cell specific genes. By comparing the entropies between cluster 1 and CD4SE by using the GNF symAtlas dataset, we found that the overall entropy of cluster 1 was larger than CD4SE (5.5 and 4.8 respectively, p < 2.2e-16), and that categorical entropy was also larger than CD4SE (10.8, and 8.95 respectively, p < 2.2e-16), implying that the genes in the cluster 1 are significantly less specific to CD4+ T cells than the genes in CD4SE. Only 66 out of 392 genes in the cluster 1 were actually CD4+ T cell specific expressions according to our definition of TSCR by gene express entropy (sensitivity = 0.14, PPV = 0.16 and F-score = 0.15).

## Conclusions

We have utilized CoreBoost to connect the HMV and TCSR patterns in CD4+ T cells. From this data we draw the following conclusions. First, we found that patterns of HMV contain sufficient information to predict TCSR target genes. The classifier we trained on HMV data successfully distinguished CD4+ T cell specific genes from housekeeping genes. Predictive HMV information was not only found in promoter regions, but also in the gene body. This finding is important because it implies the existence of multiple regulatory elements which could be marked by HMVs for TCSR. Second, we identified predictive HMV marks for CpG- and nonCpG-related genes. In promoters, H3K4me3 and H3K27ac were the most predictive HMV marks for CpG-related genes, whereas for nonCpG-related genes H3K4me3 and H3K79me3 were the most predictive. However, even if we excluded data from the most predictive HMV marks, we found that the remaining data still have sufficient predictive ability to make significant predictions for TCSR target genes. This information redundancy again points to the existence of multiple regulatory elements which could be marked by HMVs for TCSR. By carefully surveying patterns of HMV, we further propose that marking the underlying enhancers and marking intragenic alternative promoters are two potential mechanisms that could guide TCSR. Finally, we provide evidence showing that TCSR in other tissue/cell-types, as well as TCSR for non-protein coding Pol II transcripts, such as microRNA, may share TCSR HMV patterns similar to the case of CD4+ T cells. The associations between the HMV patterns and TCSR we found may be generic, as we successfully predicted genes with muscle cell specific expression, as well as microRNA genes with CD4+ T cell specific expression, by the same classifier which was trained on the HMV data of protein-coding genes in CD4+ T cells.

## Methods

### Data

The RefSeq Gene annotation track for the human genome sequence (hg18) was downloaded from the University of California Santa Cruz Genome Browser (UCSC, http://genome.ucsc.edu/). The exon information was downloaded from BioMart at Ensembl (http://www.ensembl.org/biomart/). Two gene expression data sets in human tissues were taken from the GNF symAtlas database [20]; and the GEO database (http://www.ncbi.nlm.nih.gov/geo/, GSE7307). We defined a promoter to be CpG-related if there was a CpG island located within its upstream 2 kb to downstream 500-bp region from the TSS [53]. The CpG island annotations were downloaded from the UCSC Genome Browser as well. HMV data for CD4+ T cell were retrieved from genome-wide studies of the distribution of 19 lysine or arginine histone methylations and H2A.Z histone variant [14], and mapping of 19 histone acetylation [13]. HMV data for normal human skeletal muscle myoblasts (HSMM) and K562 cell lines were retrieved from the ENCODE project [36], specifically in the Broad Institute Chip-seq dataset [24]. In addition, as part of the ENCODE project, CAGE experimental data for the K562 cell line were retrieved from the RIKEN institute [37], and DNA methylation level in K562 were retrieved from the work of Brunner and colleagues [38]. The miRNA expression profiles were retrieved from a small RNA library based sequencing atlas [52]; we used the number of clones for each miRNA cluster to represent the expression of the pri-miRNA in each tissue. The promoter of a miRNA cluster was chosen as the closet promoter predicted for the members in the cluster [19].

### Identifying tissue-specific and housekeeping transcripts

As a measurement of information content, Shannon entropy has been used for measuring the tissue-specificity of gene expression [4]. As the information content (tissue-specificity) of a distribution increases, its entropy decreases. Borrowing this concept, we measured the CD4+ T cell specific expressed gene set by the combination of the following two datasets: 1) genes that the overall gene expression entropy is smaller than 5.0 and categorical entropy less than 9 [4]; and 2) manually selected genes. From the literature, we manually selected 40 genes that play certain roles in CD4+ T cell development or maturation (see Additional file 9). The combination of the above two datasets contains 454 genes (see Additional file 9). The housekeeping genes were defined according to two criteria: 1) the overall gene expression entropy larger than 6.2 by GNF symAtlas dataset [4,20]; 2) the overall gene expression entropy larger than 8.9 by GSE7307 dataset. In total, there were 630 genes identified as housekeeping genes (see Additional file 9). The threshold for CD4+ T cell specificity was determined according to the bell shape distribution of categorical entropy (z-score > 2). The threshold for housekeeping genes was determined as the one at the turning point of the overall entropy distribution curve, which has an exponential-like shape. We also tried several thresholds surrounding values, and we retrained our model accordingly, but no significantly different results were observed.

### Definition of feature tables for promoter and gene body regions

The promoter region was defined as the region from the 6th nucleosome upstream of the transcription start site (TSS) to the 20th nucleosome downstream of the TSS. We adopted the position definitions of -2 to + 5 nucleosomes relative to the TSS used by Dustin *et al. *[2](-2[-370: -196], -1[-195: -46], +1 [-45:134], +2 [135:314], +3 [315:494], +4 [495:674] and +5 [675:859]). For the positions of other nucleosomes, we simply extended 150 bp from its immediate neighbor nucleosomes. We tried another combinations of up- and downstream nucleosome numbers to define the promoter region (from the 6th nucleosome upstream of the TSS to the 9th downstream of the TSS), but it did not change the results. To construct the feature table for a gene, HMV levels were individually calculated for each HMV type. For any given HMV type, the sum of the HMV tag numbers in a nucleosome was assigned as the HMV level on that nucleosome. The HMV feature of a gene is therefore an array containing all HMV levels of each nucleosome within the proximal promoter. Taken together, there are 40 HMV levels (including the bound level of the CCCTC-binding factor) on all 26 nucleosomes for each gene. The feature table for gene body regions is defined in the main text.

### PCA analysis was performed by using R

The sum of tags in the 4k region around the TSS ([-2k, +2K]) of all HMVs for all genes forms a matrix in which rows represent genes and columns represent HMV types. PCA analysis produced the linear combinations of columns. The first 4 principal components were chosen to form Set I and the remaining HMVs belonged to Set II.

### CoreBoost and performance evaluations

CoreBoost is a boosting technique with stumps [18]. Boosting is a supervised machine learning algorithm which combines a group of weak classifiers to form a single strong classifier [54]. Stumps are single-split decision trees with only two terminal nodes [55]. The informative features are selected by CoreBoost to build the strong classifier (for more details, see *Zhao et al. *[18]). The performance of CoreBoost classifications was evaluated by sensitivity, positive predictive values (PPV, [27]), and F-score [28], which are defined as

where TP denotes true positives, TN denotes true negatives, FP denotes false positives and FN denotes false negatives.

### 5-fold cross validation

The evaluations and the number of features for selection by CoreBoost were all obtained by 5-fold cross validations. The procedure was as follows: Given a total dataset *D*, 1) *D *was randomly partitioned into 5 subsets *D_i_*(*i *= 1,2,...,5); 2) each *D_i _*was removed exactly once from *D *and a CoreBoost classifier was trained on the remaining *80% *and tested on the removed *D_i_*; and 3) the final evaluations were the average of tests on 5 subsets. The final classifier was trained on total dataset *D*, which was used to predict the miRNAs expressed specifically in CD4+ T cells.

### The definition of control regions

We chose a position that was 50 kb upstream of any given annotated TSS as its control site. When we tried same control with 500 kb, we observed essentially the same results.

## List of abbreviations

HMV: histone modification/variation; TCSR: tissue/cell-type specific regulation; TSS: transcription start site; CAGE: Cap Analysis Gene Expression; PPV: positive prediction values; H3K4me3: histone H3 trimethylated at lysine 4; H3K4me2: histone H3 dimethylated at lysine 4; H3K4me1: histone H3 monomethylated at lysine 4; H3K27ac: histone H3 acetylated at lysine 27; H3K79me2: histone H3 dimethylated at lysine 79; H3K79me3: histone H3 trimethylated at lysine 79; H3K9ac: histone H3 acetylated at lysine 9; H3K27me3: histone H3 trimethylated at lysine 27; H3K36me3: histone H3 trimethylated at lysine 36; H4K20me1: histone H4 mono-methylated at lysine 20.

## Authors' contributions

ZZ and MZ designed the project. ZZ performed the experiment and data analysis. ZZ and MZ wrote the paper; both authors read and approved the final manuscript.

## Supplementary Material

Additional file 1 **The distribution of gene expressions across tissues**. The names of tissues are the same as those shown in the GNF symAtlas dataset. A,B,C) show the distributions for CD4SE, HK, and randomly chosen genes, respectively. Click here for file

Additional file 2 **The screeplot of the principal component analysis**. The screeplot of principal component analysis, where the *x*-axis gives the index of each principal components, and the *y*-axis gives the proportion of variance. Click here for file

Additional file 3 **Top selected HMV features by the TCSR model in gene bodies**. Top selected HMV features by the TCSR model in gene bodies. The *x*-axis shows the number of times in which an HMV feature has been selected as the top predictive feature in 100 replicates. A, B) The HMV features selected from Set I for CpG and nonCpG genes, respectively; C, D) features selected from Set II. Click here for file

Additional file 4 **Top bi-combinations of selected HMVs features by the TCSR model**. Top bi-combinations of selected HMVs features by the TCSR model. The *x*-axis shows the total number of times in which the two HMV types have been selected as the first and the second most predictive feature in 100 replicates, irrespective of nucleosomes index of the HMV features. The y-axis indicates the combinations of two HMV types in the "first_second" order. A, B) The combinations selected from Set I for CpG- and nonCpG-related promoters, respectively; C, D) combinations selected from Set II for CpG- and nonCpG-related promoters, respectively; E, F) The combinations selected from Set I for CpG- and nonCpG-related gene bodies, respectively; G, H) combinations selected from Set II for CpG- and nonCpG-related gene bodies, respectively. Click here for file

Additional file 5 **Top selected HMV features by the TCSR model**. Top selected HMV features by the TCSR model. The *x*-axis shows the number of times in which a HMV feature has been selected as the top two predictive HMV features in 100 replicates. *p *and *m*, stand for "+" and "-" strands, respectively, followed by an index of the nucleosome, either in downstream or upstream of TSS. "avg" means the average tag number of the HMV type; "body", "1stExon", and "1stIntron" means that the calculation was performed in the entire gene body region, the first exon, and the first intron region, respectively. A, B) HMVs were selected from Set I for CpG- and nonCpG-related promoters, respectively; C, D) HMVs were selected from Set II for CpG- and nonCpG-related promoters, respectively; E, F) HMVs were selected from Set I for CpG- and nonCpG-related gene bodies, respectively; C, D) HMVs were selected from Set II for CpG- and nonCpG-related gene bodies, respectively. Click here for file

Additional file 6 **The distribution of gene expression across tissues**. The distribution of gene expression across tissues. The name of tissues are the same as shown in the GNF symAtlas dataset. A) The TCSR model predicted CD4+ T cell specific genes. B) Predicted highly expressed genes in CD4+ T cells based on the gene expression activity model of Karlic *et al*. Click here for file

Additional file 7 **The HMV types highly correlated with enhancer marker H3K4me1**. All HMVs types with a Pearson's correlation coefficient compared with H3K4me1 higher than 0.2 in CD4SE genes are listed in here. The HMV names in bold font indicate they have been selected as a top predictive feature by CoreBoost at least once in 100 replicates. Click here for file

Additional file 8 **The performance of classifiers**. The classifiers were trained by the HMV profile subset of CD4+ T cell, in which H3K4me3, H3K4me2, H3K79me3, and H3K27ac were not included. Averages and errors are given as the mean and standard deviation, respectively, from 100 replicates. The performances were measured by applying the classifiers on protein-coding genes in CD4+ T cells. The significances of comparison between the performance of CoreBoost trained on features in those regions and control regions are indicated by symbols next to each number No symbol indicates p-value < 1e-5; * indicates p-value < 1e-2 and > = 1e-5; indicates p-value > 1e-2. Click here for file

Additional  file 9 **Gene name list**. This file lists the CD4+ T cell specific and housekeeping protein-coding genes and miRNA genes. Click here for file
