# Supplementary figures and images for: Histone modification profiles are predictive for tissue/cell-type specific expression of both protein-coding and microRNA genes
Source: BMC Bioinformatics. 2011 May 14;12:155. doi: 10.1186/1471-2105-12-155 (PMC3120700; doi:10.1186/1471-2105-12-155)

The expression distribution among tissues

A

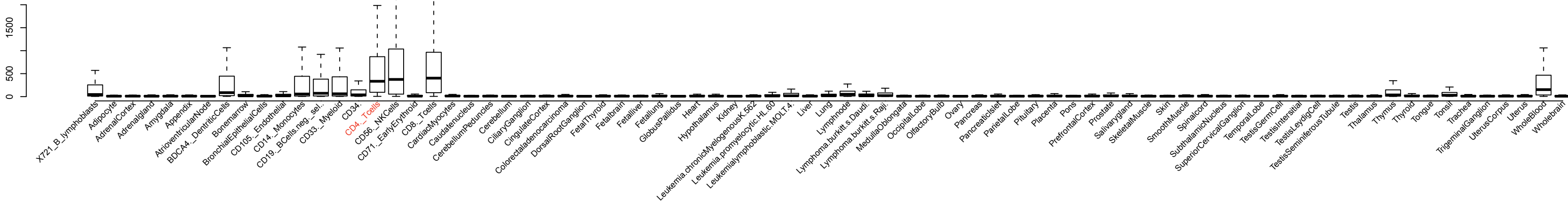

B

Expression level

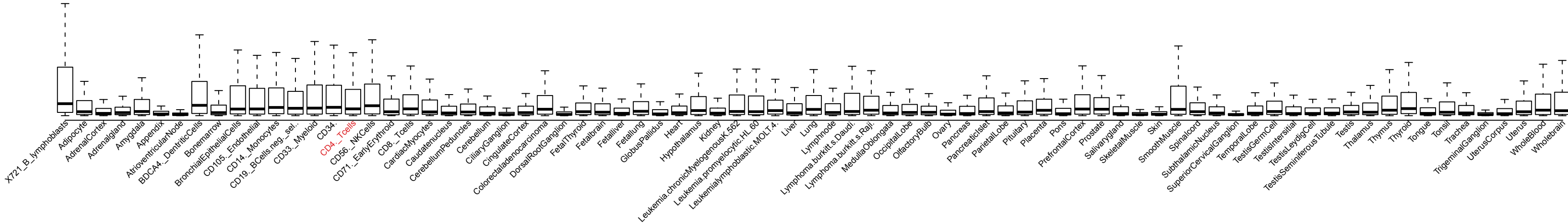

C

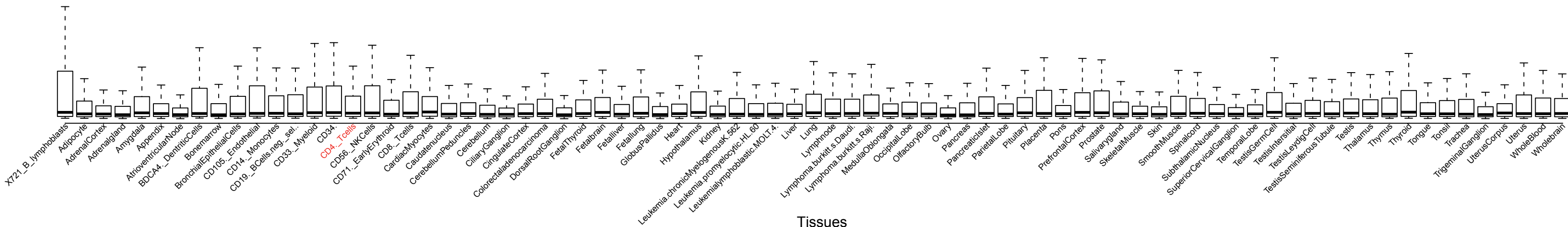

Tissues

Supplement: Additional file 1 — The distribution of gene expressions across tissues. The names of tissues are the same as those shown in the GNF symAtlas dataset. A,B,C) show the distributions for CD4SE, HK, and randomly chosen genes, respectively. [file 1471-2105-12-155-S1.PDF]

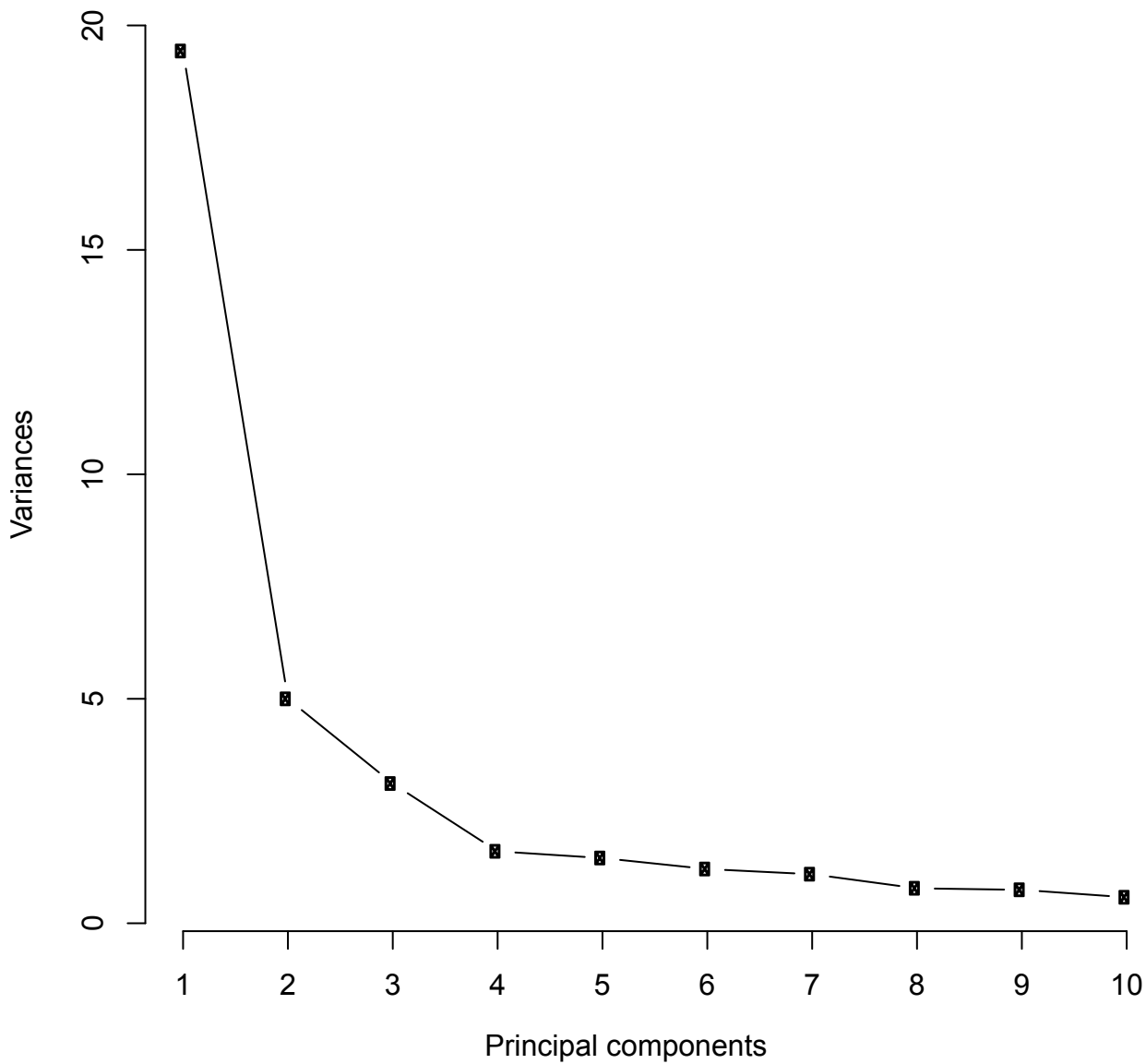

Supplement: Additional file 2 — The screeplot of the principal component analysis. The screeplot of principal component analysis, where the x-axis gives the index of each principal components, and the y-axis gives the proportion of variance. [file 1471-2105-12-155-S2.PDF]

A

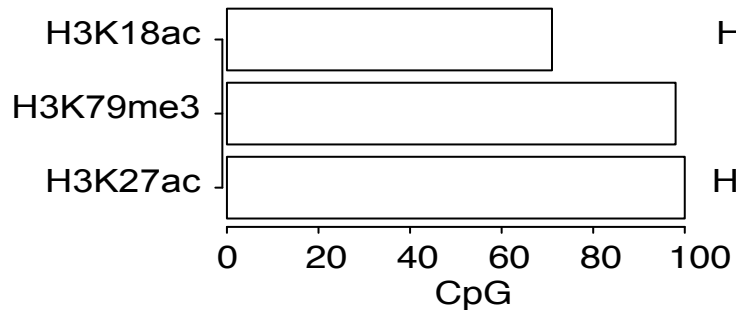

B

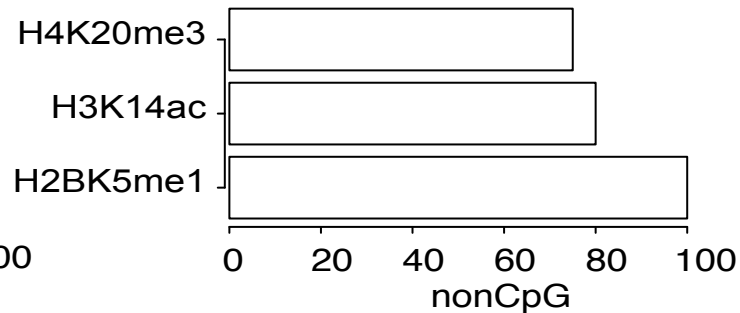

C

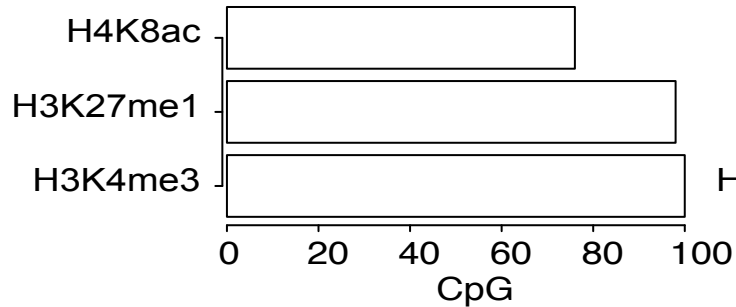

D

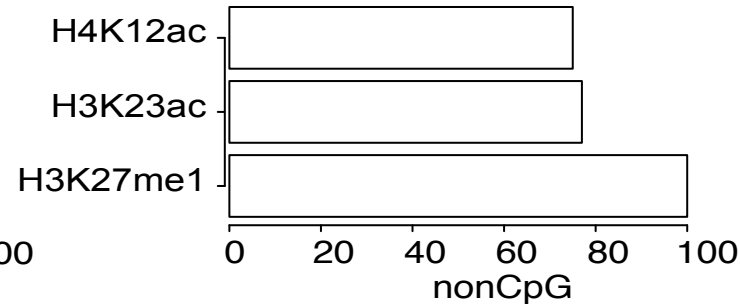

Supplement: Additional file 3 — Top selected HMV features by the TCSR model in gene bodies. Top selected HMV features by the TCSR model in gene bodies. The x-axis shows the number of times in which an HMV feature has been selected as the top predictive feature in 100 replicates. A, B) The HMV features selected from Set I for CpG and nonCpG genes, respectively; C, D) features selected from Set II. [file 1471-2105-12-155-S3.PDF]

**A**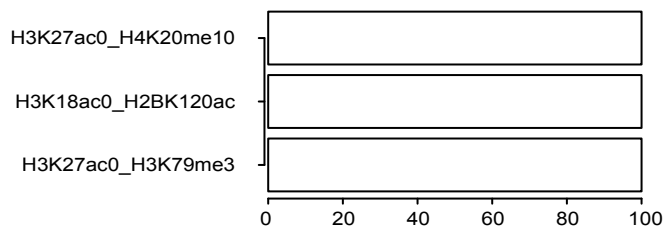**B**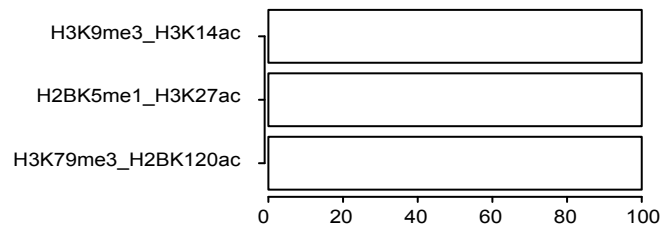**C**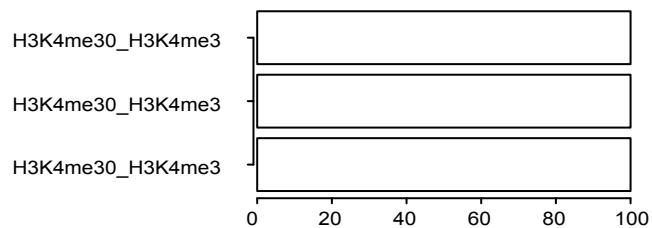**D**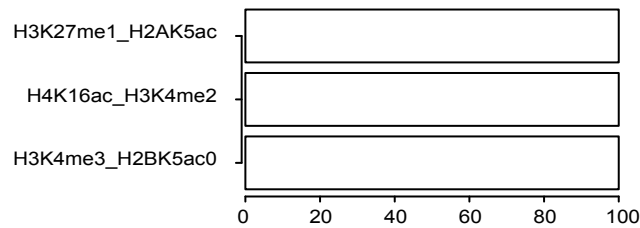**E**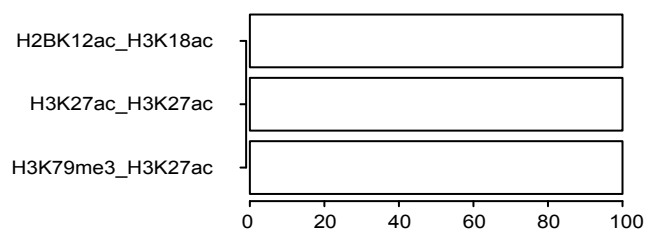**F**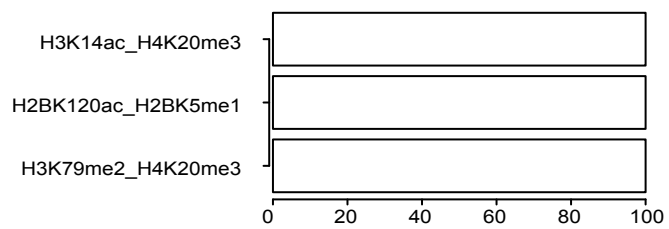**G**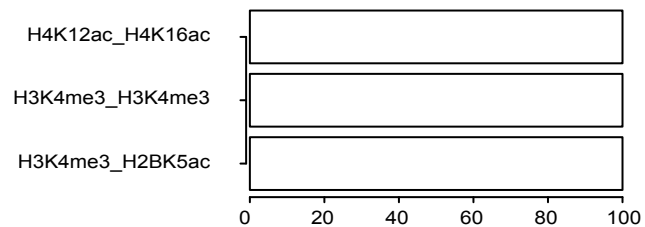**H**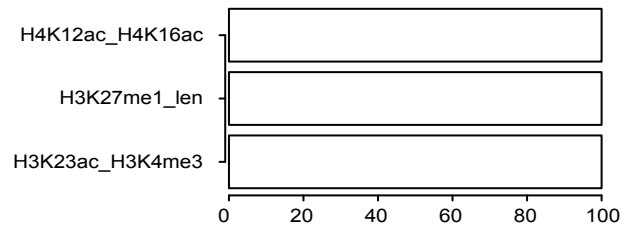

CpG

nonCpG

Supplement: Additional file 4 — Top bi-combinations of selected HMVs features by the TCSR model. Top bi-combinations of selected HMVs features by the TCSR model. The x-axis shows the total number of times in which the two HMV types have been selected as the first and the second most predictive feature in 100 replicates, irrespective of nucleosomes index of the HMV features. The y-axis indicates the combinations of two HMV types in the "first_second" order. A, B) The combinations selected from Set I for CpG- and nonCpG-related promoters, respectively; C, D) combinations selected from Set II for CpG- and nonCpG-related promoters, respectively; E, F) The combinations selected from Set I for CpG- and nonCpG-related gene bodies, respectively; G, H) combinations selected from Set II for CpG- and nonCpG-related gene bodies, respectively. [file 1471-2105-12-155-S4.PDF]

**A**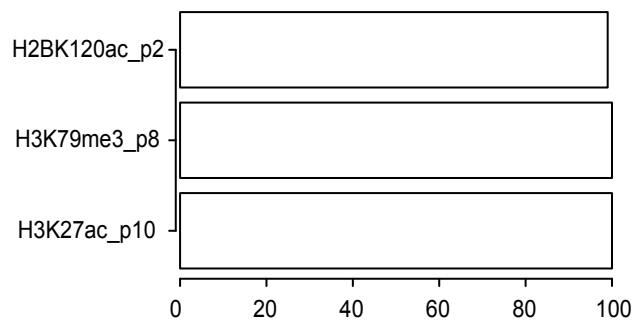**B**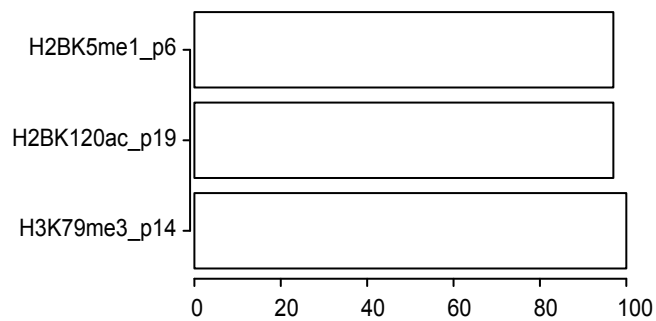**C**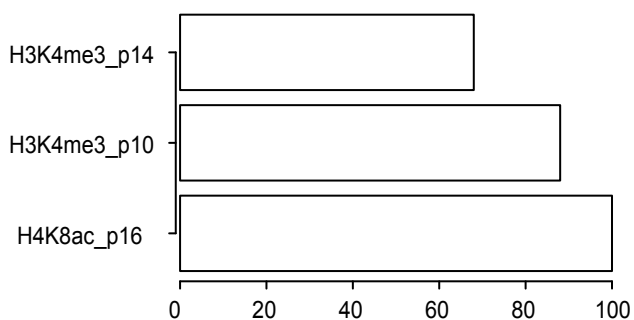**D**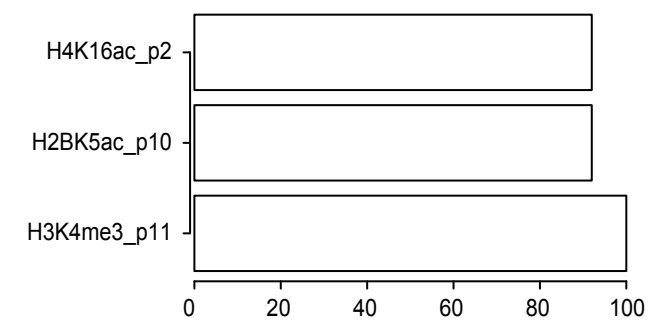**E**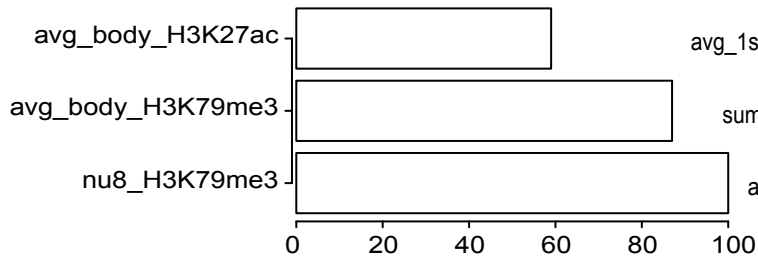**F**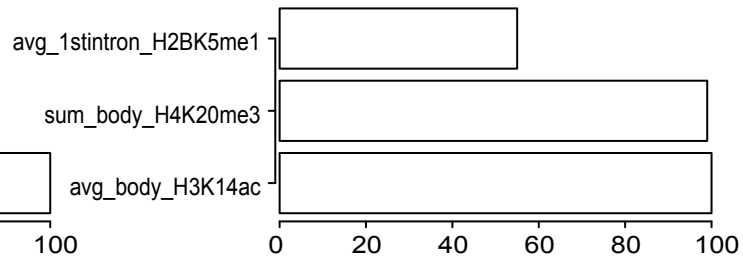**G**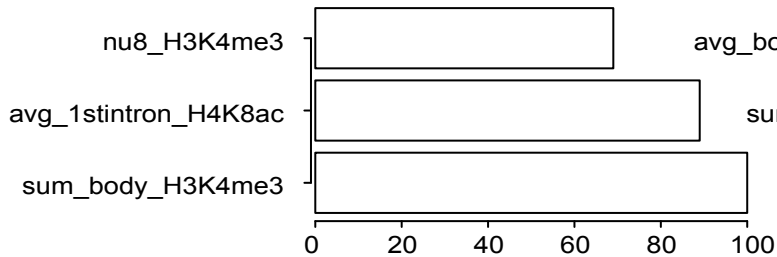**H**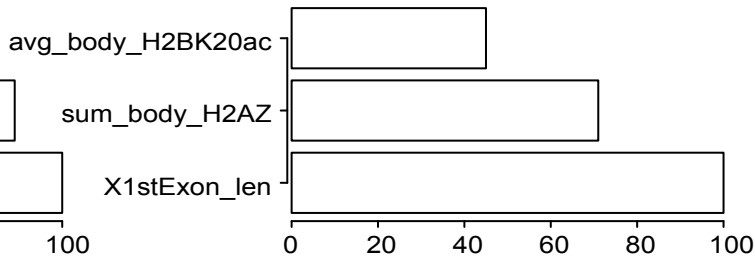

CpG

nonCpG

Supplement: Additional file 5 — Top selected HMV features by the TCSR model. Top selected HMV features by the TCSR model. The x-axis shows the number of times in which a HMV feature has been selected as the top two predictive HMV features in 100 replicates. p and m, stand for "+" and "-" strands, respectively, followed by an index of the nucleosome, either in downstream or upstream of TSS. "avg" means the average tag number of the HMV type; "body", "1stExon", and "1stIntron" means that the calculation was performed in the entire gene body region, the first exon, and the first intron region, respectively. A, B) HMVs were selected from Set I for CpG- and nonCpG-related promoters, respectively; C, D) HMVs were selected from Set II for CpG- and nonCpG-related promoters, respectively; E, F) HMVs were selected from Set I for CpG- and nonCpG-related gene bodies, respectively; C, D) HMVs were selected from Set II for CpG- and nonCpG-related gene bodies, respectively. [file 1471-2105-12-155-S5.PDF]

The expression distribution among tissues

A

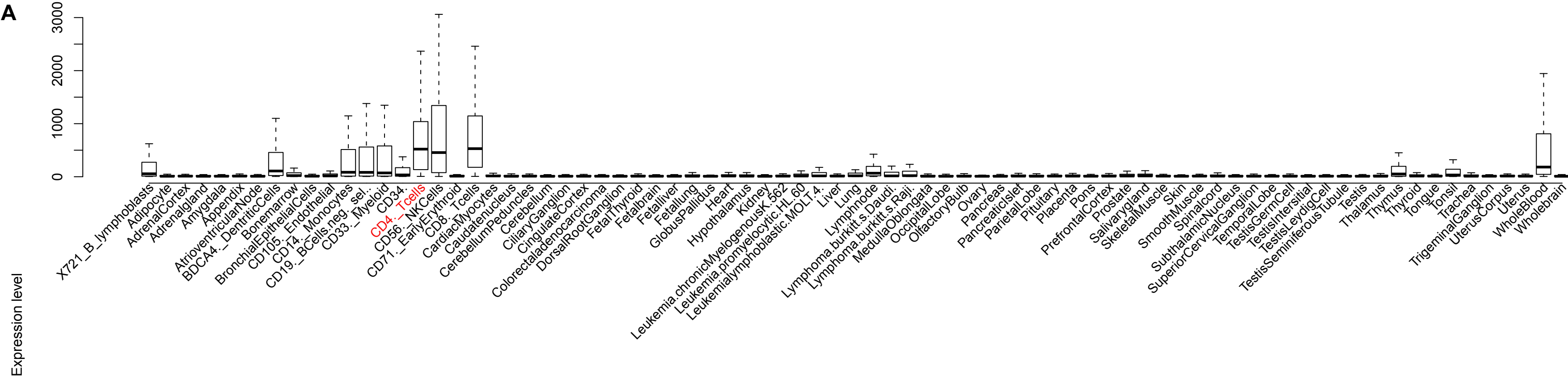

B

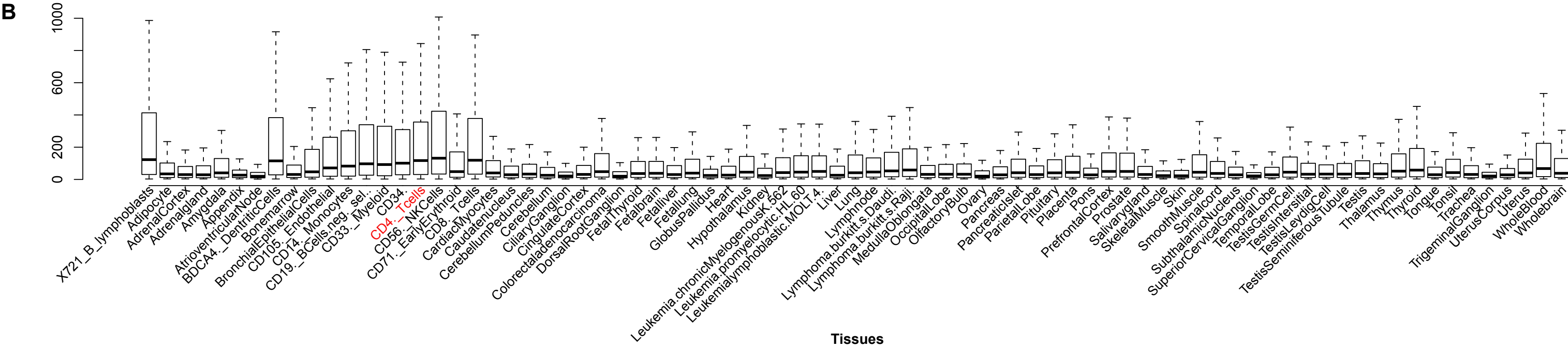

Supplement: Additional file 6 — The distribution of gene expression across tissues. The distribution of gene expression across tissues. The name of tissues are the same as shown in the GNF symAtlas dataset. A) The TCSR model predicted CD4+ T cell specific genes. B) Predicted highly expressed genes in CD4+ T cells based on the gene expression activity model of Karlic et al. [file 1471-2105-12-155-S6.PDF]
