# Supplementary material for: Histone modification profiles are predictive for tissue/cell-type specific expression of both protein-coding and microRNA genes
Source: BMC Bioinformatics. 2011 May 14;12:155. doi: 10.1186/1471-2105-12-155 (PMC3120700; doi:10.1186/1471-2105-12-155)
Supplement: Additional file 7 — The HMV types highly correlated with enhancer marker H3K4me1. All HMVs types with a Pearson's correlation coefficient compared with H3K4me1 higher than 0.2 in CD4SE genes are listed in here. The HMV names in bold font indicate they have been selected as a top predictive feature by CoreBoost at least once in 100 replicates. [file 1471-2105-12-155-S7.DOC]

|  | Median | Mean |
| --- | --- | --- |
| **H2AK5ac** | 0.2741 | 0.2846 |
| **H2BK120ac** | 0.2045 | 0.2143 |
| H2BK20ac | 0.2225 | 0.2377 |
| **H4K16ac** | 0.2144 | 0.2231 |
| **H4K5ac** | 0.2923 | 0.2874 |
| **H4K8ac** | 0.2687 | 0.2431 |
| **H2BK5me1** | 0.5311 | 0.4902 |
| H3K27me1 | 0.4378 | 0.3909 |
| **H3K36me1** | 0.2705 | 0.2620 |
| **H3K36me3** | 0.2080 | 0.2047 |
| **H3K4me2** | 0.3740 | 0.3530 |
| **H3K79me1** | 0.3652 | 0.3348 |
| **H3K9me1** | 0.4605 | 0.4442 |
| **H3R2me1** | 0.3077 | 0.2880 |
| **H4K20me1** | 0.3655 | 0.3226 |

**Table S1.** The HMV types that highly correlated to enhancer marker H3K4me1. All HMVs types with Pearson’s correlation coefficient to H3K4me1 higher than 0.2 in CD4SE genes are listed in here. The HMV names in bold font indicate it has been selected as top predictive feature by CoreBoost at least once in 100 replicates.
