# Supplementary material for: Histone modification profiles are predictive for tissue/cell-type specific expression of both protein-coding and microRNA genes
Source: BMC Bioinformatics. 2011 May 14;12:155. doi: 10.1186/1471-2105-12-155 (PMC3120700; doi:10.1186/1471-2105-12-155)
Supplement: Additional file 8 — The performance of classifiers. The classifiers were trained by the HMV profile subset of CD4+ T cell, in which H3K4me3, H3K4me2, H3K79me3, and H3K27ac were not included. Averages and errors are given as the mean and standard deviation, respectively, from 100 replicates. The performances were measured by applying the classifiers on protein-coding genes in CD4+ T cells. The significances of comparison between the performance of CoreBoost trained on features in those regions and control regions are indicated by symbols next to each number No symbol indicates p-value < 1e-5; * indicates p-value < 1e-2 and > = 1e-5; indicates p-value > 1e-2. [file 1471-2105-12-155-S8.DOC]

| **HMV groups** |  |  | **Sensitivity** | **PPV** | **F-Score** |
| --- | --- | --- | --- | --- | --- |
| **Set I** | Promoter | CpG | 0.670±0.021 | 0.724±0.022 | 0.696±0.014 |
|  |  | non-CpG | 0.780±0.039 | 0.729±0.032 | 0.752±0.018 |
|  | Body | CpG | 0.706±0.018 | 0.753±0.020 | 0.728±0.013 |
|  |  | non-CpG | 0.810±0.029 | 0.788±0.030 | 0.798±0.020 |
| **Set II** | Promoter | CpG | 0.677±0.021 | 0.710±0.022 | 0.693±0.015 |
|  |  | non-CpG | 0.805±0.034 | 0.742±0.029 | 0.771±0.018 |
|  | Body | CpG | 0.693±0.019 | 0.727±0.020 | 0.709±0.013 |
|  |  | non-CpG | 0.834±0.025 | 0.782±0.025 | 0.807±0.017 |

**Table S2**. The performance of classifiers. The classifiers were trained by CD4+ T cell’s HMV profiles subset, in which H3K4me3, H3K4me2, H3K79me3, and H3K27ac were not included. Averages and errors are given as the mean and standard deviation, respectively, from 100 replicates. The performance were measured by applying the classifiers on protein-coding genes in CD4+ T cell. The significances of comparison between the performance of Coreboost trained on features in those regions and control regions are indicated by symbols next to each number. No symbol, p-value < 1e-5; *, p-value < 1e-2 and >= 1e-5; † , p-value > 1e-2.
