# Supplementary material for: Histone modification profiles are predictive for tissue/cell-type specific expression of both protein-coding and microRNA genes
Source: BMC Bioinformatics. 2011 May 14;12:155. doi: 10.1186/1471-2105-12-155 (PMC3120700; doi:10.1186/1471-2105-12-155)
Supplement: Additional file 9 — Gene name list. This file lists the CD4+ T cell specific and housekeeping protein-coding genes and miRNA genes. [file 1471-2105-12-155-S9.DOC]

SI dataset 1. Manually selected CD4+ T cell related genes.

SI dataset 2. CD4+ T cell specific expressed genes.

SI dataset 3. Housekeeping genes.

SI dataset 4. CD4+ T cell specific expressed miRNA.

SI dataset 5. Housekeeping miRNAs.

SI dataset 6. The HMV types in Set I and Set II.

SI dataset 1. . Manually selected CD4+ T cell related genes.

| EntrezGene | GeneID | PMID | Description |
| --- | --- | --- | --- |
| PRKCE | 5581 | 11175263 | protein kinase C, epsilon |
| PIK3R4 | 30849 | 19587117 | phosphoinositide-3-kinase, regulatory subunit 4 |
| CD4 | 920 | NBK10757* | CD4 molecule |
| PRKCZ | 5590 | 11175263 | protein kinase C, zeta |
| GATA3 | 2625 | 20484083 | GATA binding protein 3 |
| PRKCQ | 5588 | 11175263 | protein kinase C, theta |
| PIK3C2B | 5287 | 19587117 | phosphoinositide-3-kinase, class 2, beta polypeptide |
| RUNX3 | 864 | 19165227 | runt-related transcription factor 3 |
| PRKCH | 5583 | 11175263 | protein kinase C, eta |
| PIK3R1 | 5295 | 19587117 | phosphoinositide-3-kinase, regulatory subunit 1 (alpha) |
| MAF | 4094 | 20042469 | v-maf musculoaponeurotic fibrosarcoma oncogene homolog (avian) |
| PIK3R5 | 23533 | 19587117 | phosphoinositide-3-kinase, regulatory subunit 5 |
| CD80 | 941 | NBK10757* | CD80 molecule |
| PIK3CG | 5294 | 19587117 | phosphoinositide-3-kinase, catalytic, gamma polypeptide |
| NFKB1 | 4790 | 20103781 | nuclear factor of kappa light polypeptide gene enhancer in B-cells 1 |
| ICOS | 29851 | 20116985 | inducible T-cell co-stimulator |
| PIK3C2G | 5288 | 19587117 | phosphoinositide-3-kinase, class 2, gamma polypeptide |
| PIK3CA | 5290 | 19587117 | phosphoinositide-3-kinase, catalytic, alpha polypeptide |
| NFAT5 | 10725 | 20103781 | nuclear factor of activated T-cells 5, tonicity-responsive |
| ZBTB7B | 51043 | 17878336 | zinc finger and BTB domain containing 7B |
| PRKCI | 5584 | 11175263 | protein kinase C, iota |
| CD28 | 940 | NBK10757* | CD28 molecule |
| PIK3CB | 5291 | 19587117 | phosphoinositide-3-kinase, catalytic, beta polypeptide |
| PIK3R6 | 146850 | 19587117 | phosphoinositide-3-kinase, regulatory subunit 6 |
| STAT6 | 6778 | 19535633 | signal transducer and activator of transcription 6, interleukin-4 induced |
| PIK3C3 | 5289 | 19587117 | phosphoinositide-3-kinase, class 3 |
| STAT4 | 6775 | 19923468 | signal transducer and activator of transcription 4 |
| PIK3R2 | 5296 | 19587117 | phosphoinositide-3-kinase, regulatory subunit 2 (beta) |
| CD86 | 942 | NBK10757* | CD86 molecule |
| CTCF | 10664 | NBK10757* | CCCTC-binding factor (zinc finger protein) |
| PRKCA | 5578 | 11175263 | protein kinase C, alpha |
| PRKCD | 5580 | 11175263 | protein kinase C, delta |
| STAT1 | 6772 | NBK10757* | signal transducer and activator of transcription 1, 91kDa |
| RUNX1 | 861 | 19165227 | runt-related transcription factor 1 |
| VAV1 | 7409 | 19060239 | vav 1 guanine nucleotide exchange factor |
| HLX | 3142 | 14688316 | H2.0-like homeobox |
| RUNX2 | 860 | 19165227 | runt-related transcription factor 2 |
| PIK3R3 | 8503 | 19587117 | phosphoinositide-3-kinase, regulatory subunit 3 (gamma) |
| PIK3C2A | 5286 | 19587117 | phosphoinositide-3-kinase, class 2, alpha polypeptide |
| MYB | 4602 | 20484083 | v-myb myeloblastosis viral oncogene homolog (avian) |

*NCBI Bookshelf ID

SI dataset 2. CD4+ T cell specific expressed genes.

| EntrezGene | GeneID | Description |
| --- | --- | --- |
| RHOH | 399 | ras homolog gene family, member H |
| TXK | 7294 | TXK tyrosine kinase |
| AASDH | 132949 | aminoadipate-semialdehyde dehydrogenase |
| YTHDC1 | 91746 | YTH domain containing 1 |
| MRPL1 | 65008 | mitochondrial ribosomal protein L1 |
| PLAC8 | 51316 | placenta-specific 8 |
| MAEA | 10296 | macrophage erythroblast attacher |
| SEMA4D | 10507 | sema domain, immunoglobulin domain (Ig), transmembrane domain (TM) and short cytoplasmic domain, (semaphorin) 4D |
| HLA-A | 3105 | major histocompatibility complex, class I, A |
| MICB | 4277 | MHC class I polypeptide-related sequence B |
| LTB | 4050 | lymphotoxin beta (TNF superfamily, member 3) |
| NCR3 | 259197 | natural cytotoxicity triggering receptor 3 |
| HLA-DOB | 3112 | major histocompatibility complex, class II, DO beta |
| TAP2 | 6891 | transporter 2, ATP-binding cassette, sub-family B (MDR/TAP) |
| PSMB8 | 5696 | proteasome (prosome, macropain) subunit, beta type, 8 (large multifunctional peptidase 7) |
| PSMB9 | 5698 | proteasome (prosome, macropain) subunit, beta type, 9 (large multifunctional peptidase 2) |
| HLA-DMB | 3109 | major histocompatibility complex, class II, DM beta |
| HLA-DMA | 3108 | major histocompatibility complex, class II, DM alpha |
| DYX4 | 3111 | dyslexia susceptibility 4 |
| HLA-DPA1 | 3113 | major histocompatibility complex, class II, DP alpha 1 |
| HLA-DPB1 | 3115 | major histocompatibility complex, class II, DP beta 1 |
| PIK3CG | 5294 | phosphoinositide-3-kinase, catalytic, gamma polypeptide |
| TMEM106B | 54664 | transmembrane protein 106B |
| SUSD3 | 203328 | sushi domain containing 3 |
| ZC3HAV1 | 56829 | zinc finger CCCH-type, antiviral 1 |
| TRBV7-9 | 6957 | T cell receptor beta variable 7-9 |
| TRBC1 | 28639 | T cell receptor beta constant 1 |
| TRBC2 | 28638 | T cell receptor beta constant 2 |
| GIMAP7 | 168537 | GTPase, IMAP family member 7 |
| GIMAP6 | 474344 | GTPase, IMAP family member 6 |
| GIMAP2 | 26157 | GTPase, IMAP family member 2 |
| GIMAP1 | 170575 | GTPase, IMAP family member 1 |
| GIMAP5 | 55340 | GTPase, IMAP family member 5 |
| CARD11 | 84433 | caspase recruitment domain family, member 11 |
| TRGC2 | 6965 | T cell receptor gamma constant 2 |
| PTPLAD2 | 401494 | protein tyrosine phosphatase-like A domain containing 2 |
| MYO1G | 64005 | myosin IG |
| UPP1 | 7378 | uridine phosphorylase 1 |
| IKZF1 | 10320 | IKAROS family zinc finger 1 (Ikaros) |
| NSUN5C | 260294 | NOL1/NOP2/Sun domain family, member 5C |
| NSUN5 | 55695 | NOL1/NOP2/Sun domain family, member 5 |
| NSUN5B | 155400 | NOL1/NOP2/Sun domain family, member 5B |
| PION | 54103 | pigeon homolog (Drosophila) |
| AZGP1 | 563 | alpha-2-glycoprotein 1, zinc-binding |
| PVRIG | 79037 | poliovirus receptor related immunoglobulin domain containing |
| PILRB | 29990 | paired immunoglobin-like type 2 receptor beta |
| BIN3 | 55909 | bridging integrator 3 |
| CHMP7 | 91782 | CHMP family, member 7 |
| LEPROTL1 | 23484 | leptin receptor overlapping transcript-like 1 |
| PDE7A | 5150 | phosphodiesterase 7A |
| MYBL1 | 4603 | v-myb myeloblastosis viral oncogene homolog (avian)-like 1 |
| AMMECR1 | 9949 | Alport syndrome, mental retardation, midface hypoplasia and elliptocytosis chromosomal region gene 1 |
| PAG1 | 55824 | phosphoprotein associated with glycosphingolipid microdomains 1 |
| MFHAS1 | 9258 | malignant fibrous histiocytoma amplified sequence 1 |
| MSL3 | 10943 | male-specific lethal 3 homolog (Drosophila) |
| AKNA | 80709 | AT-hook transcription factor |
| C9orf142 | 286257 | chromosome 9 open reading frame 142 |
| METT11D1 | 64745 | methyltransferase 11 domain containing 1 |
| METTL3 | 56339 | methyltransferase like 3 |
| SEPT6 | 23157 | septin 6 |
| TRDC | 6955 | T cell receptor delta constant |
| TRAJ17 | 28738 | T cell receptor alpha joining 17 |
| TRAC | 28755 | T cell receptor alpha constant |
| NFATC4 | 4776 | nuclear factor of activated T-cells, cytoplasmic, calcineurin-dependent 4 |
| SDR39U1 | 56948 | short chain dehydrogenase/reductase family 39U, member 1 |
| GZMH | 2999 | granzyme H (cathepsin G-like 2, protein h-CCPX) |
| MTMR15 | 22909 | myotubularin related protein 15 |
| RASGRP1 | 10125 | RAS guanyl releasing protein 1 (calcium and DAG-regulated) |
| PLA2G4B | 8681 | phospholipase A2, group IVB (cytosolic) |
| RORA | 6095 | RAR-related orphan receptor A |
| SH2D1A | 4068 | SH2 domain protein 1A |
| USP3 | 9960 | ubiquitin specific peptidase 3 |
| PSTPIP1 | 9051 | proline-serine-threonine phosphatase interacting protein 1 |
| IL16 | 3603 | interleukin 16 (lymphocyte chemoattractant factor) |
| ISG20 | 3669 | interferon stimulated exonuclease gene 20kDa |
| TMEM204 | 79652 | transmembrane protein 204 |
| NME3 | 4832 | non-metastatic cells 3, protein expressed in |
| PRO0461 | 5170 | hypothetical LOC652276 |
| PRKCB | 5579 | protein kinase C, beta |
| IL4R | 3566 | interleukin 4 receptor |
| LAT | 27040 | linker for activation of T cells |
| IL32 | 9235 | interleukin 32 |
| CORO1A | 11151 | coronin, actin binding protein, 1A |
| SEPT1 | 1731 | septin 1 |
| ADCY7 | 113 | adenylate cyclase 7 |
| CYLD | 1540 | cylindromatosis (turban tumor syndrome) |
| AKTIP | 64400 | AKT interacting protein |
| NLRC5 | 84166 | NLR family, CARD domain containing 5 |
| CBFB | 865 | core-binding factor, beta subunit |
| CTCF | 10664 | CCCTC-binding factor (zinc finger protein) |
| PSMB10 | 5699 | proteasome (prosome, macropain) subunit, beta type, 10 |
| DPEP2 | 64174 | dipeptidase 2 |
| NFATC3 | 4775 | nuclear factor of activated T-cells, cytoplasmic, calcineurin-dependent 3 |
| NFAT5 | 10725 | nuclear factor of activated T-cells 5, tonicity-responsive |
| MAF | 4094 | v-maf musculoaponeurotic fibrosarcoma oncogene homolog (avian) |
| COTL1 | 23406 | coactosin-like 1 (Dictyostelium) |
| ANKRD11 | 29123 | ankyrin repeat domain 11 |
| ZNF276 | 92822 | zinc finger protein 276 |
| ATF7IP2 | 80063 | activating transcription factor 7 interacting protein 2 |
| ARHGEF6 | 9459 | Rac/Cdc42 guanine nucleotide exchange factor (GEF) 6 |
| EVI2B | 2124 | ecotropic viral integration site 2B |
| EVI2A | 2123 | ecotropic viral integration site 2A |
| CCL5 | 6352 | chemokine (C-C motif) ligand 5 |
| GSDMB | 55876 | gasdermin B |
| CCR7 | 1236 | chemokine (C-C motif) receptor 7 |
| STAT5B | 6777 | signal transducer and activator of transcription 5B |
| TBX21 | 30009 | T-box 21 |
| CDK5RAP3 | 80279 | CDK5 regulatory subunit associated protein 3 |
| SKAP1 | 8631 | src kinase associated phosphoprotein 1 |
| FAM117A | 81558 | family with sequence similarity 117, member A |
| RSAD1 | 55316 | radical S-adenosyl methionine domain containing 1 |
| ARRB2 | 409 | arrestin, beta 2 |
| NLRP1 | 22861 | NLR family, pyrin domain containing 1 |
| RPS6KB1 | 6198 | ribosomal protein S6 kinase, 70kDa, polypeptide 1 |
| C9orf72 | 203228 | chromosome 9 open reading frame 72 |
| PRKCA | 5578 | protein kinase C, alpha |
| ABCA5 | 23461 | ATP-binding cassette, sub-family A (ABC1), member 5 |
| ACAP1 | 9744 | ArfGAP with coiled-coil, ankyrin repeat and PH domains 1 |
| CD300A | 11314 | CD300a molecule |
| RAB37 | 326624 | RAB37, member RAS oncogene family |
| CYTH1 | 9267 | cytohesin 1 |
| USP36 | 57602 | ubiquitin specific peptidase 36 |
| ENGASE | 64772 | endo-beta-N-acetylglucosaminidase |
| C17orf68 | 80169 | chromosome 17 open reading frame 68 |
| CD7 | 924 | CD7 molecule |
| PIK3R6 | 146850 | phosphoinositide-3-kinase, regulatory subunit 6 |
| PIK3R5 | 23533 | phosphoinositide-3-kinase, regulatory subunit 5 |
| PIK3C3 | 5289 | phosphoinositide-3-kinase, class 3 |
| ZFP161 | 7541 | zinc finger protein 161 homolog (mouse) |
| POLI | 11201 | polymerase (DNA directed) iota |
| NFATC1 | 4772 | nuclear factor of activated T-cells, cytoplasmic, calcineurin-dependent 1 |
| CCDC130 | 81576 | coiled-coil domain containing 130 |
| CD97 | 976 | CD97 molecule |
| PKN1 | 5585 | protein kinase N1 |
| KLF2 | 10365 | Kruppel-like factor 2 (lung) |
| PIK3R2 | 5296 | phosphoinositide-3-kinase, regulatory subunit 2 (beta) |
| GZMM | 3004 | granzyme M (lymphocyte met-ase 1) |
| TMEM149 | 79713 | transmembrane protein 149 |
| LIN37 | 55957 | lin-37 homolog (C. elegans) |
| MAP4K1 | 11184 | mitogen-activated protein kinase kinase kinase kinase 1 |
| GMFG | 9535 | glia maturation factor, gamma |
| TGFB1 | 7040 | transforming growth factor, beta 1 |
| ARHGEF1 | 9138 | Rho guanine nucleotide exchange factor (GEF) 1 |
| ZNF224 | 7767 | zinc finger protein 224 |
| EMP3 | 2014 | epithelial membrane protein 3 |
| CD37 | 951 | CD37 molecule |
| FLT3LG | 2323 | fms-related tyrosine kinase 3 ligand |
| FCGRT | 2217 | Fc fragment of IgG, receptor, transporter, alpha |
| IRF3 | 3661 | interferon regulatory factor 3 |
| NKG7 | 4818 | natural killer cell group 7 sequence |
| VAV1 | 7409 | vav 1 guanine nucleotide exchange factor |
| CD209 | 30835 | CD209 molecule |
| MYO1F | 4542 | myosin IF |
| HMHA1 | 23526 | histocompatibility (minor) HA-1 |
| C19orf66 | 55337 | chromosome 19 open reading frame 66 |
| ICAM3 | 3385 | intercellular adhesion molecule 3 |
| KRI1 | 65095 | KRI1 homolog (S. cerevisiae) |
| SLC44A2 | 57153 | solute carrier family 44, member 2 |
| SIRPG | 55423 | signal-regulatory protein gamma |
| CST7 | 8530 | cystatin F (leukocystatin) |
| CDC25B | 994 | cell division cycle 25 homolog B (S. pombe) |
| SAMHD1 | 25939 | SAM domain and HD domain 1 |
| PLCG1 | 5335 | phospholipase C, gamma 1 |
| EIF2S3 | 1968 | eukaryotic translation initiation factor 2, subunit 3 gamma, 52kDa |
| NFATC2 | 4773 | nuclear factor of activated T-cells, cytoplasmic, calcineurin-dependent 2 |
| STX16 | 8675 | syntaxin 16 |
| SLMO2 | 51012 | slowmo homolog 2 (Drosophila) |
| LIME1 | 54923 | Lck interacting transmembrane adaptor 1 |
| C21orf45 | 54069 | chromosome 21 open reading frame 45 |
| IFNAR2 | 3455 | interferon (alpha, beta and omega) receptor 2 |
| TMEM50B | 757 | transmembrane protein 50B |
| RUNX1 | 861 | runt-related transcription factor 1 |
| MX2 | 4600 | myxovirus (influenza virus) resistance 2 (mouse) |
| MX1 | 4599 | myxovirus (influenza virus) resistance 1, interferon-inducible protein p78 (mouse) |
| LOC100134052 | 53347 | similar to ubiquitin associated and SH3 domain containing, A |
| PRKX | 5613 | protein kinase, X-linked |
| UBE2G2 | 7327 | ubiquitin-conjugating enzyme E2G 2 (UBC7 homolog, yeast) |
| ITGB2 | 3689 | integrin, beta 2 (complement component 3 receptor 3 and 4 subunit) |
| PRMT2 | 3275 | protein arginine methyltransferase 2 |
| RNF149 | 284996 | ring finger protein 149 |
| IL18RAP | 8807 | interleukin 18 receptor accessory protein |
| PTPN4 | 5775 | protein tyrosine phosphatase, non-receptor type 4 (megakaryocyte) |
| TRIB2 | 28951 | tribbles homolog 2 (Drosophila) |
| CXCR4 | 7852 | chemokine (C-X-C motif) receptor 4 |
| ARHGAP15 | 55843 | Rho GTPase activating protein 15 |
| NMI | 9111 | N-myc (and STAT) interactor |
| CYTIP | 9595 | cytohesin 1 interacting protein |
| ITGA4 | 3676 | integrin, alpha 4 (antigen CD49D, alpha 4 subunit of VLA-4 receptor) |
| STAT1 | 6772 | signal transducer and activator of transcription 1, 91kDa |
| STAT4 | 6775 | signal transducer and activator of transcription 4 |
| STK17B | 9262 | serine/threonine kinase 17b |
| CASP8 | 841 | caspase 8, apoptosis-related cysteine peptidase |
| CD28 | 940 | CD28 molecule |
| ICOS | 29851 | inducible T-cell co-stimulator |
| SP110 | 3431 | SP110 nuclear body protein |
| SP140L | 93349 | SP140 nuclear body protein-like |
| INPP5D | 3635 | inositol polyphosphate-5-phosphatase, 145kDa |
| ARL4C | 10123 | ADP-ribosylation factor-like 4C |
| AGBL5 | 60509 | ATP/GTP binding protein-like 5 |
| LBH | 81606 | limb bud and heart development homolog (mouse) |
| BIRC6 | 57448 | baculoviral IAP repeat-containing 6 |
| NUDCP2 | 6432 | nuclear distribution gene C homolog (A. nidulans) pseudogene 2 |
| ZFP36L2 | 678 | zinc finger protein 36, C3H type-like 2 |
| PRKCE | 5581 | protein kinase C, epsilon |
| ARHGAP25 | 9938 | Rho GTPase activating protein 25 |
| TIA1 | 7072 | TIA1 cytotoxic granule-associated RNA binding protein |
| RETSAT | 54884 | retinol saturase (all-trans-retinol 13,14-reductase) |
| GNLY | 10578 | granulysin |
| CD8A | 925 | CD8a molecule |
| CD8B | 926 | CD8b molecule |
| RPIA | 22934 | ribose 5-phosphate isomerase A |
| MAL | 4118 | mal, T-cell differentiation protein |
| DUSP2 | 1844 | dual specificity phosphatase 2 |
| ZAP70 | 7535 | zeta-chain (TCR) associated protein kinase 70kDa |
| NFKBIZ | 64332 | nuclear factor of kappa light polypeptide gene enhancer in B-cells inhibitor, zeta |
| TRAT1 | 50852 | T cell receptor associated transmembrane adaptor 1 |
| CD80 | 941 | CD80 molecule |
| HCLS1 | 3059 | hematopoietic cell-specific Lyn substrate 1 |
| CD86 | 942 | CD86 molecule |
| PIK3R4 | 30849 | phosphoinositide-3-kinase, regulatory subunit 4 |
| ACAD11 | 27031 | acyl-Coenzyme A dehydrogenase family, member 11 |
| PIK3CB | 5291 | phosphoinositide-3-kinase, catalytic, beta polypeptide |
| RASA2 | 5922 | RAS p21 protein activator 2 |
| GPR171 | 29909 | G protein-coupled receptor 171 |
| MBNL1 | 4154 | muscleblind-like (Drosophila) |
| PRKCI | 5584 | protein kinase C, iota |
| PIK3CA | 5290 | phosphoinositide-3-kinase, catalytic, alpha polypeptide |
| RBM26 | 64062 | RNA binding motif protein 26 |
| SYTL1 | 84958 | synaptotagmin-like 1 |
| C1orf38 | 9473 | chromosome 1 open reading frame 38 |
| CD247 | 919 | CD247 molecule |
| EIF4B | 1975 | eukaryotic translation initiation factor 4B |
| ITGB7 | 3695 | integrin, beta 7 |
| PCTK2 | 5128 | PCTAIRE protein kinase 2 |
| PDCD4 | 27250 | programmed cell death 4 (neoplastic transformation inhibitor) |
| MR1 | 3140 | major histocompatibility complex, class I-related |
| PTPRC | 5788 | protein tyrosine phosphatase, receptor type, C |
| TRAF3IP3 | 80342 | TRAF3 interacting protein 3 |
| LOC100133233 | 100133233 | hypothetical protein LOC100133233 |
| SIVA1 | 10572 | SIVA1, apoptosis-inducing factor |
| N4BP2L2 | 10443 | NEDD4 binding protein 2-like 2 |
| KLF12 | 11278 | Kruppel-like factor 12 |
| C12orf35 | 55196 | chromosome 12 open reading frame 35 |
| ATHL1 | 80162 | ATH1, acid trehalase-like 1 (yeast) |
| SIGIRR | 59307 | single immunoglobulin and toll-interleukin 1 receptor (TIR) domain |
| IRF7 | 3665 | interferon regulatory factor 7 |
| TNFRSF4 | 7293 | tumor necrosis factor receptor superfamily, member 4 |
| RUNX3 | 864 | runt-related transcription factor 3 |
| SLAMF1 | 6504 | signaling lymphocytic activation molecule family member 1 |
| CD48 | 962 | CD48 molecule |
| ARHGAP30 | 257106 | Rho GTPase activating protein 30 |
| CCDC88C | 440193 | coiled-coil domain containing 88C |
| PIK3C2A | 5286 | phosphoinositide-3-kinase, class 2, alpha polypeptide |
| BIRC3 | 330 | baculoviral IAP repeat-containing 3 |
| C10orf54 | 64115 | chromosome 10 open reading frame 54 |
| PIK3CD | 5293 | phosphoinositide-3-kinase, catalytic, delta polypeptide |
| XCL2 | 6846 | chemokine (C motif) ligand 2 |
| XCL1 | 6375 | chemokine (C motif) ligand 1 |
| GNG2 | 54331 | guanine nucleotide binding protein (G protein), gamma 2 |
| PTGER2 | 5732 | prostaglandin E receptor 2 (subtype EP2), 53kDa |
| NCKAP1L | 3071 | NCK-associated protein 1-like |
| DYRK2 | 8445 | dual-specificity tyrosine-(Y)-phosphorylation regulated kinase 2 |
| IFNG | 3458 | interferon, gamma |
| ETS1 | 2113 | v-ets erythroblastosis virus E26 oncogene homolog 1 (avian) |
| CD2 | 914 | CD2 molecule |
| PPOX | 5498 | protoporphyrinogen oxidase |
| IL2RG | 3561 | interleukin 2 receptor, gamma (severe combined immunodeficiency) |
| TMEM63A | 9725 | transmembrane protein 63A |
| ERP27 | 121506 | endoplasmic reticulum protein 27 |
| ARHGDIB | 397 | Rho GDP dissociation inhibitor (GDI) beta |
| PIK3C2G | 5288 | phosphoinositide-3-kinase, class 2, gamma polypeptide |
| SLC38A1 | 81539 | solute carrier family 38, member 1 |
| PAN2 | 9924 | PAN2 poly(A) specific ribonuclease subunit homolog (S. cerevisiae) |
| STAT6 | 6778 | signal transducer and activator of transcription 6, interleukin-4 induced |
| CARD17 | 834 | caspase recruitment domain family, member 17 |
| BCL11B | 64919 | B-cell CLL/lymphoma 11B (zinc finger protein) |
| SELL | 6402 | selectin L |
| RIN3 | 79890 | Ras and Rab interactor 3 |
| ERCC5 | 2073 | excision repair cross-complementing rodent repair deficiency, complementation group 5 |
| CD4 | 920 | CD4 molecule |
| PTPN6 | 5777 | protein tyrosine phosphatase, non-receptor type 6 |
| PDE3B | 5140 | phosphodiesterase 3B, cGMP-inhibited |
| CD6 | 923 | CD6 molecule |
| GATA3 | 2625 | GATA binding protein 3 |
| AMY2B | 280 | amylase, alpha 2B (pancreatic) |
| AMY2A | 279 | amylase, alpha 2A (pancreatic) |
| AMY1A | 276 | amylase, alpha 1A (salivary) |
| AMY1B | 277 | amylase, alpha 1B (salivary) |
| AMY1C | 278 | amylase, alpha 1C (salivary) |
| LCP1 | 3936 | lymphocyte cytosolic protein 1 (L-plastin) |
| BIN2 | 51411 | bridging integrator 2 |
| EML3 | 256364 | echinoderm microtubule associated protein like 3 |
| CTSW | 1521 | cathepsin W |
| SART1 | 9092 | squamous cell carcinoma antigen recognized by T cells |
| ATM | 472 | ataxia telangiectasia mutated |
| KLF6 | 1316 | Kruppel-like factor 6 |
| ABLIM1 | 3983 | actin binding LIM protein 1 |
| AKIRIN1 | 79647 | akirin 1 |
| GALT | 2592 | galactose-1-phosphate uridylyltransferase |
| ZBTB25 | 7597 | zinc finger and BTB domain containing 25 |
| IL11RA | 3590 | interleukin 11 receptor, alpha |
| MCI2 | 241 | Myocardial infarction, susceptiblity to, 2 |
| GPR18 | 2841 | G protein-coupled receptor 18 |
| GPR183 | 1880 | G protein-coupled receptor 183 |
| KLRG1 | 10219 | killer cell lectin-like receptor subfamily G, member 1 |
| C11orf21 | 29125 | chromosome 11 open reading frame 21 |
| FERMT3 | 83706 | fermitin family homolog 3 (Drosophila) |
| RASGRP2 | 10235 | RAS guanyl releasing protein 2 (calcium and DAG-regulated) |
| IL10RA | 3587 | interleukin 10 receptor, alpha |
| AMICA1 | 120425 | adhesion molecule, interacts with CXADR antigen 1 |
| CD3E | 916 | CD3e molecule, epsilon (CD3-TCR complex) |
| CD3D | 915 | CD3d molecule, delta (CD3-TCR complex) |
| CD3G | 917 | CD3g molecule, gamma (CD3-TCR complex) |
| MARCH8 | 220972 | membrane-associated ring finger (C3HC4) 8 |
| PRKCZ | 5590 | protein kinase C, zeta |
| ZBTB40 | 9923 | zinc finger and BTB domain containing 40 |
| PKN2 | 5586 | protein kinase N2 |
| GBP2 | 2634 | guanylate binding protein 2, interferon-inducible |
| GBP5 | 115362 | guanylate binding protein 5 |
| CD53 | 963 | CD53 molecule |
| DENND2D | 79961 | DENN/MADD domain containing 2D |
| C1orf162 | 128346 | chromosome 1 open reading frame 162 |
| PRPF3 | 9129 | PRP3 pre-mRNA processing factor 3 homolog (S. cerevisiae) |
| C1orf56 | 54964 | chromosome 1 open reading frame 56 |
| TNFAIP8L2 | 79626 | tumor necrosis factor, alpha-induced protein 8-like 2 |
| PBXIP1 | 57326 | pre-B-cell leukemia homeobox interacting protein 1 |
| ZBTB7B | 51043 | zinc finger and BTB domain containing 7B |
| RASSF5 | 83593 | Ras association (RalGDS/AF-6) domain family member 5 |
| FAIM3 | 9214 | Fas apoptotic inhibitory molecule 3 |
| FAM113B | 91523 | family with sequence similarity 113, member B |
| ARHGAP9 | 64333 | Rho GTPase activating protein 9 |
| TRIM22 | 10346 | tripartite motif-containing 22 |
| LPXN | 9404 | leupaxin |
| TRAF5 | 7188 | TNF receptor-associated factor 5 |
| GPR65 | 8477 | G protein-coupled receptor 65 |
| KLRB1 | 3820 | killer cell lectin-like receptor subfamily B, member 1 |
| CLEC2D | 29121 | C-type lectin domain family 2, member D |
| CD69 | 969 | CD69 molecule |
| KLRF1 | 51348 | killer cell lectin-like receptor subfamily F, member 1 |
| KLRD1 | 3824 | killer cell lectin-like receptor subfamily D, member 1 |
| KLRK1 | 22914 | killer cell lectin-like receptor subfamily K, member 1 |
| KLRC2 | 3822 | killer cell lectin-like receptor subfamily C, member 2 |
| KLRC1 | 3821 | killer cell lectin-like receptor subfamily C, member 1 |
| USP9Y | 8287 | ubiquitin specific peptidase 9, Y-linked |
| PRF1 | 5551 | perforin 1 (pore forming protein) |
| FOXO1 | 2308 | forkhead box O1 |
| NARG1L | 79612 | NMDA receptor regulated 1-like |
| TCIRG1 | 10312 | T-cell, immune regulator 1, ATPase, H+ transporting, lysosomal V0 subunit A3 |
| PIK3C2B | 5287 | phosphoinositide-3-kinase, class 2, beta polypeptide |
| PRKCH | 5583 | protein kinase C, eta |
| JARID1D | 8284 | jumonji, AT rich interactive domain 1D |
| PIK3R3 | 8503 | phosphoinositide-3-kinase, regulatory subunit 3 (gamma) |
| SMYD3 | 64754 | SET and MYND domain containing 3 |
| LAPTM5 | 7805 | lysosomal multispanning membrane protein 5 |
| LCK | 3932 | lymphocyte-specific protein tyrosine kinase |
| TBC1D4 | 9882 | TBC1 domain family, member 4 |
| ARGLU1 | 55082 | arginine and glutamate rich 1 |
| CD27 | 939 | CD27 molecule |
| SRGN | 5552 | serglycin |
| SAI1 | 6275 | suppression of anchorage independence 1 |
| MAP3K9 | 4293 | mitogen-activated protein kinase kinase kinase 9 |
| PCNX | 22990 | pecanex homolog (Drosophila) |
| PHF11 | 51131 | PHD finger protein 11 |
| SFRS5 | 6430 | splicing factor, arginine/serine-rich 5 |
| DGKA | 1606 | diacylglycerol kinase, alpha 80kDa |
| TBC1D10C | 374403 | TBC1 domain family, member 10C |
| PTPRCAP | 5790 | protein tyrosine phosphatase, receptor type, C-associated protein |
| ADD3 | 120 | adducin 3 (gamma) |
| LDLRAP1 | 26119 | low density lipoprotein receptor adaptor protein 1 |
| LOC100134000 | 57134 | similar to mannosidase, alpha, class 1C, member 1 |
| CD52 | 1043 | CD52 molecule |
| ZDHHC18 | 84243 | zinc finger, DHHC-type containing 18 |
| PRKCQ | 5588 | protein kinase C, theta |
| MGEA5 | 10724 | meningioma expressed antigen 5 (hyaluronidase) |
| TXNIP | 10628 | thioredoxin interacting protein |
| HLX | 3142 | H2.0-like homeobox |
| GCH1 | 2643 | GTP cyclohydrolase 1 |
| MAX | 4149 | MYC associated factor X |
| CECR1 | 51816 | cat eye syndrome chromosome region, candidate 1 |
| PIK3IP1 | 113791 | phosphoinositide-3-kinase interacting protein 1 |
| IL2RB | 3560 | interleukin 2 receptor, beta |
| RAC2 | 5880 | ras-related C3 botulinum toxin substrate 2 (rho family, small GTP binding protein Rac2) |
| CYTH4 | 27128 | cytohesin 4 |
| MFNG | 4242 | MFNG O-fucosylpeptide 3-beta-N-acetylglucosaminyltransferase |
| DDX17 | 10521 | DEAD (Asp-Glu-Ala-Asp) box polypeptide 17 |
| SGSM3 | 27352 | small G protein signaling modulator 3 |
| FAM118A | 55007 | family with sequence similarity 118, member A |
| TBC1D22A | 25771 | TBC1 domain family, member 22A |
| TUBGCP6 | 85378 | tubulin, gamma complex associated protein 6 |
| LPCAT1 | 79888 | lysophosphatidylcholine acyltransferase 1 |
| ENSG00000145779 |  | Tumor necrosis factor, alpha-induced protein 8 (Head and neck tumor and metastasis-related protein)(TNF- induced protein GG2-1)(SCC-S2)(NF-kappa-B-inducible DED- containing protein)(NDED)(MDC-3.13) [Source:UniProtKB/Swiss-Prot;Acc:O95379] |
| FNIP1 | 51735 | folliculin interacting protein 1 |
| IRF1 | 3659 | interferon regulatory factor 1 |
| IL5 | 3567 | interleukin 5 (colony-stimulating factor, eosinophil) |
| RAD50 | 10111 | RAD50 homolog (S. cerevisiae) |
| IL13 | 3596 | interleukin 13 |
| IL4 | 3565 | interleukin 4 |
| TCF7 | 6932 | transcription factor 7 (T-cell specific, HMG-box) |
| PCYOX1L | 78991 | prenylcysteine oxidase 1 like |
| CD74 | 972 | CD74 molecule, major histocompatibility complex, class II invariant chain |
| CCDC69 | 26112 | coiled-coil domain containing 69 |
| ITK | 3702 | IL2-inducible T-cell kinase |
| DOCK2 | 1794 | dedicator of cytokinesis 2 |
| ENSG00000043462 |  | Lymphocyte cytosolic protein 2 (SH2 domain- containing leukocyte protein of 76 kDa)(SLP-76 tyrosine phosphoprotein)(SLP76) [Source:UniProtKB/Swiss- Prot;Acc:Q13094] |
| STK10 | 6793 | serine/threonine kinase 10 |
| RNF44 | 22838 | ring finger protein 44 |
| CLK4 | 57396 | CDC-like kinase 4 |
| IL7R | 3575 | interleukin 7 receptor |
| ENSG00000082074 |  | FYN-binding protein (FYN-T-binding protein)(FYB- 120/130)(p120/p130)(SLP-76-associated phosphoprotein)(SLAP-130)(Adhesion and degranulation promoting adaptor protein)(ADAP) [Source:UniProtKB/Swiss- Prot;Acc:O15117] |
| PTGER4 | 5734 | prostaglandin E receptor 4 (subtype EP4) |
| GZMK | 3003 | granzyme K (granzyme 3; tryptase II) |
| GZMA | 3001 | granzyme A (granzyme 1, cytotoxic T-lymphocyte-associated serine esterase 3) |
| ANKRD55 | 79722 | ankyrin repeat domain 55 |
| PPWD1 | 23398 | peptidylprolyl isomerase domain and WD repeat containing 1 |
| PIK3R1 | 5295 | phosphoinositide-3-kinase, regulatory subunit 1 (alpha) |
| MTRR | 4552 | 5-methyltetrahydrofolate-homocysteine methyltransferase reductase |
| ERAP2 | 64167 | endoplasmic reticulum aminopeptidase 2 |
| AIM1 | 202 | absent in melanoma 1 |
| MED23 | 9439 | mediator complex subunit 23 |
| VNN2 | 8875 | vanin 2 |
| MYB | 4602 | v-myb myeloblastosis viral oncogene homolog (avian) |
| TNFAIP3 | 7128 | tumor necrosis factor, alpha-induced protein 3 |
| HECA | 51696 | headcase homolog (Drosophila) |
| ULBP2 | 80328 | UL16 binding protein 2 |
| ULBP1 | 80329 | UL16 binding protein 1 |
| TAGAP | 117289 | T-cell activation RhoGTPase activating protein |
| FAM65B | 9750 | family with sequence similarity 65, member B |
| HFE | 3077 | hemochromatosis |
| BTN3A2 | 11118 | butyrophilin, subfamily 3, member A2 |
| BTN3A3 | 10384 | butyrophilin, subfamily 3, member A3 |
| BTN2A1 | 11120 | butyrophilin, subfamily 2, member A1 |
| DEF6 | 50619 | differentially expressed in FDCP 6 homolog (mouse) |
| SUPT3H | 8464 | suppressor of Ty 3 homolog (S. cerevisiae) |
| RUNX2 | 860 | runt-related transcription factor 2 |
| BACH2 | 60468 | BTB and CNC homology 1, basic leucine zipper transcription factor 2 |
| PRKACG | 5568 | protein kinase, cAMP-dependent, catalytic, gamma |
| SATB1 | 6304 | SATB homeobox 1 |
| CRBN | 51185 | cereblon |
| CX3CR1 | 1524 | chemokine (C-X3-C motif) receptor 1 |
| NKTR | 4820 | natural killer-tumor recognition sequence |
| SNRK | 54861 | SNF related kinase |
| CCR2 | 729230 | chemokine (C-C motif) receptor 2 |
| DAG1 | 1605 | dystroglycan 1 (dystrophin-associated glycoprotein 1) |
| C9orf95 | 54981 | chromosome 9 open reading frame 95 |
| TUSC4 | 10641 | tumor suppressor candidate 4 |
| PRKCD | 5580 | protein kinase C, delta |
| ATXN7 | 6314 | ataxin 7 |
| NFKB1 | 4790 | nuclear factor of kappa light polypeptide gene enhancer in B-cells 1 |
| LEF1 | 51176 | lymphoid enhancer-binding factor 1 |
| CCDC109B | 55013 | coiled-coil domain containing 109B |
| IL2 | 3558 | interleukin 2 |
| INPP4B | 8821 | inositol polyphosphate-4-phosphatase, type II, 105kDa |
| KIAA0746 | 23231 | KIAA0746 protein |
| RBPJ | 3516 | recombination signal binding protein for immunoglobulin kappa J region |

SI dataset 3. Housekeeping genes.

| EntrezGene | GeneID | Description |
| --- | --- | --- |
| C5orf24 | 134553 | chromosome 5 open reading frame 24 |
| ZNF664 | 144348 | zinc finger protein 664 |
| ZSCAN22 | 342945 | zinc finger and SCAN domain containing 22 |
| DCTN2 | 10540 | dynactin 2 (p50) |
| MTHFSD | 64779 | methenyltetrahydrofolate synthetase domain containing |
| PELI3 | 246330 | pellino homolog 3 (Drosophila) |
| UGCGL2 | 55757 | UDP-glucose ceramide glucosyltransferase-like 2 |
| GCC1 | 79571 | GRIP and coiled-coil domain containing 1 |
| C8orf83 | 286144 | chromosome 8 open reading frame 83 |
| ZNF608 | 57507 | zinc finger protein 608 |
| ZNF71 | 58491 | zinc finger protein 71 |
| FXC1 | 26515 | fracture callus 1 homolog (rat) |
| NCRNA00171 | 80862 | non-protein coding RNA 171 |
| CWF19L2 | 143884 | CWF19-like 2, cell cycle control (S. pombe) |
| SH2B1 | 25970 | SH2B adaptor protein 1 |
| TTLL4 | 9654 | tubulin tyrosine ligase-like family, member 4 |
| UBIAD1 | 29914 | UbiA prenyltransferase domain containing 1 |
| PML | 5371 | promyelocytic leukemia |
| WDR33 | 55339 | WD repeat domain 33 |
| MAN2C1 | 4123 | mannosidase, alpha, class 2C, member 1 |
| BAT2L | 84726 | HLA-B associated transcript 2-like |
| JMJD2A | 9682 | jumonji domain containing 2A |
| ZNF212 | 7988 | zinc finger protein 212 |
| OGG1 | 4968 | 8-oxoguanine DNA glycosylase |
| BRPF3 | 27154 | bromodomain and PHD finger containing, 3 |
| LOC100133918 | 100131704 | similar to nuclear receptor co-repressor 1 |
| GSTCD | 79807 | glutathione S-transferase, C-terminal domain containing |
| ZNF629 | 23361 | zinc finger protein 629 |
| RING1 | 6015 | ring finger protein 1 |
| ZNF434 | 54925 | zinc finger protein 434 |
| ANKFY1 | 51479 | ankyrin repeat and FYVE domain containing 1 |
| OGFOD2 | 79676 | 2-oxoglutarate and iron-dependent oxygenase domain containing 2 |
| C7orf64 | 84060 | chromosome 7 open reading frame 64 |
| TP53BP1 | 7158 | tumor protein p53 binding protein 1 |
| CSNK1E | 1454 | casein kinase 1, epsilon |
| VTI1A | 143187 | vesicle transport through interaction with t-SNAREs homolog 1A (yeast) |
| BRWD1 | 54014 | bromodomain and WD repeat domain containing 1 |
| RBM12B | 389677 | RNA binding motif protein 12B |
| UNKL | 64718 | unkempt homolog (Drosophila)-like |
| INVS | 27130 | inversin |
| MDM2 | 4193 | Mdm2 p53 binding protein homolog (mouse) |
| ATF7 | 11016 | activating transcription factor 7 |
| CYHR1 | 50626 | cysteine/histidine-rich 1 |
| RHBDD1 | 84236 | rhomboid domain containing 1 |
| PIK3R4 | 30849 | phosphoinositide-3-kinase, regulatory subunit 4 |
| ZNF550 | 162972 | zinc finger protein 550 |
| ARNT | 405 | aryl hydrocarbon receptor nuclear translocator |
| MARCH9 | 92979 | membrane-associated ring finger (C3HC4) 9 |
| EXD3 | 54932 | exonuclease 3'-5' domain containing 3 |
| MAGIX | 79917 | MAGI family member, X-linked |
| ZNF337 | 26152 | zinc finger protein 337 |
| CCRN4L | 25819 | CCR4 carbon catabolite repression 4-like (S. cerevisiae) |
| CUL7 | 9820 | cullin 7 |
| C1orf107 | 27042 | chromosome 1 open reading frame 107 |
| CSRNP2 | 81566 | cysteine-serine-rich nuclear protein 2 |
| PRDM5 | 11107 | PR domain containing 5 |
| MLLT10 | 8028 | myeloid/lymphoid or mixed-lineage leukemia (trithorax homolog, Drosophila); translocated to, 10 |
| RNF185 | 91445 | ring finger protein 185 |
| SCAPER | 49855 | S-phase cyclin A-associated protein in the ER |
| FER | 2241 | fer (fps/fes related) tyrosine kinase |
| NENF | 29937 | neuron derived neurotrophic factor |
| UCK1 | 83549 | uridine-cytidine kinase 1 |
| MLL3 | 58508 | myeloid/lymphoid or mixed-lineage leukemia 3 |
| C22orf29 | 79680 | chromosome 22 open reading frame 29 |
| TUBD1 | 51174 | tubulin, delta 1 |
| CCDC57 | 284001 | coiled-coil domain containing 57 |
| SFT2D3 | 84826 | SFT2 domain containing 3 |
| SNX19 | 399979 | sorting nexin 19 |
| ZNF500 | 26048 | zinc finger protein 500 |
| RSF1 | 51773 | remodeling and spacing factor 1 |
| SMARCD1 | 6602 | SWI/SNF related, matrix associated, actin dependent regulator of chromatin, subfamily d, member 1 |
| BCORL1 | 63035 | BCL6 co-repressor-like 1 |
| IPP | 3652 | intracisternal A particle-promoted polypeptide |
| ATG10 | 83734 | ATG10 autophagy related 10 homolog (S. cerevisiae) |
| ZNF506 | 440515 | zinc finger protein 506 |
| LRRC37B | 114659 | leucine rich repeat containing 37B |
| ZFP28 | 140612 | zinc finger protein 28 homolog (mouse) |
| ZSCAN2 | 54993 | zinc finger and SCAN domain containing 2 |
| KIAA1429 | 25962 | KIAA1429 |
| MTHFR | 4524 | 5,10-methylenetetrahydrofolate reductase (NADPH) |
| TGFBRAP1 | 9392 | transforming growth factor, beta receptor associated protein 1 |
| SLC12A4 | 6560 | solute carrier family 12 (potassium/chloride transporters), member 4 |
| ZFPL1 | 7542 | zinc finger protein-like 1 |
| SMPD1 | 6609 | sphingomyelin phosphodiesterase 1, acid lysosomal |
| MRI1 | 84245 | methylthioribose-1-phosphate isomerase homolog (S. cerevisiae) |
| ACTR8 | 93973 | ARP8 actin-related protein 8 homolog (yeast) |
| POLDIP3 | 84271 | polymerase (DNA-directed), delta interacting protein 3 |
| EEFSEC | 60678 | eukaryotic elongation factor, selenocysteine-tRNA-specific |
| TBC1D20 | 128637 | TBC1 domain family, member 20 |
| EPS15L1 | 58513 | epidermal growth factor receptor pathway substrate 15-like 1 |
| ST7L | 54879 | suppression of tumorigenicity 7 like |
| STK35 | 140901 | serine/threonine kinase 35 |
| EDA | 1896 | ectodysplasin A |
| ZNF551 | 90233 | zinc finger protein 551 |
| PARP16 | 54956 | poly (ADP-ribose) polymerase family, member 16 |
| ASB7 | 140460 | ankyrin repeat and SOCS box-containing 7 |
| ENGASE | 64772 | endo-beta-N-acetylglucosaminidase |
| ZNF544 | 27300 | zinc finger protein 544 |
| TBC1D9B | 23061 | TBC1 domain family, member 9B (with GRAM domain) |
| LOC100272228 |  |  |
| ATP9B | 374868 | ATPase, class II, type 9B |
| CSPP1 | 79848 | centrosome and spindle pole associated protein 1 |
| ZNF19 | 7567 | zinc finger protein 19 |
| ZNF343 | 79175 | zinc finger protein 343 |
| ZNF717 |  | zinc finger protein 717 |
| ARHGEF11 | 9826 | Rho guanine nucleotide exchange factor (GEF) 11 |
| ZNF592 | 9640 | zinc finger protein 592 |
| INO80 | 54617 | INO80 homolog (S. cerevisiae) |
| SMG5 | 23381 | Smg-5 homolog, nonsense mediated mRNA decay factor (C. elegans) |
| ZZEF1 | 23140 | zinc finger, ZZ-type with EF-hand domain 1 |
| EDC3 | 80153 | enhancer of mRNA decapping 3 homolog (S. cerevisiae) |
| SRCAP | 10847 | Snf2-related CREBBP activator protein |
| NUFIP1 | 26747 | nuclear fragile X mental retardation protein interacting protein 1 |
| MFSD11 | 79157 | major facilitator superfamily domain containing 11 |
| TTC5 | 91875 | tetratricopeptide repeat domain 5 |
| ZNF202 | 7753 | zinc finger protein 202 |
| ZNF555 | 148254 | zinc finger protein 555 |
| C9orf6 | 54942 | chromosome 9 open reading frame 6 |
| UBQLN4 | 56893 | ubiquilin 4 |
| GBA2 | 57704 | glucosidase, beta (bile acid) 2 |
| ZNF630 | 7569 | zinc finger protein 630 |
| FBXL20 | 84961 | F-box and leucine-rich repeat protein 20 |
| ARID1B | 57492 | AT rich interactive domain 1B (SWI1-like) |
| PLA2G6 | 8398 | phospholipase A2, group VI (cytosolic, calcium-independent) |
| BRAF | 673 | v-raf murine sarcoma viral oncogene homolog B1 |
| ZNF678 | 339500 | zinc finger protein 678 |
| MAP1D | 254042 | methionine aminopeptidase 1D |
| ZBTB40 | 9923 | zinc finger and BTB domain containing 40 |
| RPL37A | 6168 | ribosomal protein L37a |
| C3orf75 | 54859 | chromosome 3 open reading frame 75 |
| PIGL | 9487 | phosphatidylinositol glycan anchor biosynthesis, class L |
| ZBTB45 | 84878 | zinc finger and BTB domain containing 45 |
| TAS2R14 | 50840 | taste receptor, type 2, member 14 |
| PLEKHA8 | 84725 | pleckstrin homology domain containing, family A (phosphoinositide binding specific) member 8 |
| TMEM41A | 90407 | transmembrane protein 41A |
| MTRF1L | 54516 | mitochondrial translational release factor 1-like |
| NCOA2 | 10499 | nuclear receptor coactivator 2 |
| PGBD2 | 267002 | piggyBac transposable element derived 2 |
| WDR89 | 112840 | WD repeat domain 89 |
| ENSG00000206062 |  | CRYBB2P1 protein [Source:UniProtKB/TrEMBL;Acc:Q4G0T4] |
| C9orf7 | 11094 | chromosome 9 open reading frame 7 |
| CCBL1 | 883 | cysteine conjugate-beta lyase, cytoplasmic |
| CSNK1G1 | 53944 | casein kinase 1, gamma 1 |
| ZNF614 | 9668 | zinc finger protein 614 |
| PIGO | 84720 | phosphatidylinositol glycan anchor biosynthesis, class O |
| CHD2 | 1106 | chromodomain helicase DNA binding protein 2 |
| HMGXB3 | 22993 | HMG box domain containing 3 |
| ZNF710 | 374655 | zinc finger protein 710 |
| KIAA0195 | 9772 | KIAA0195 |
| LOC644096 | 644096 | hypothetical protein LOC644096 |
| KIAA0562 | 9731 | KIAA0562 |
| GPR75 | 10936 | G protein-coupled receptor 75 |
| MRPS25 | 64432 | mitochondrial ribosomal protein S25 |
| ZNF621 | 285268 | zinc finger protein 621 |
| OFCC1 | 266553 | orofacial cleft 1 candidate 1 |
| ZNF490 | 57474 | zinc finger protein 490 |
| DIP2A | 23181 | DIP2 disco-interacting protein 2 homolog A (Drosophila) |
| PHF20L1 | 51105 | PHD finger protein 20-like 1 |
| MAPK8 | 5599 | mitogen-activated protein kinase 8 |
| PQLC2 | 54896 | PQ loop repeat containing 2 |
| DNAJC16 | 23341 | DnaJ (Hsp40) homolog, subfamily C, member 16 |
| C15orf44 | 81556 | chromosome 15 open reading frame 44 |
| NME6 | 10201 | non-metastatic cells 6, protein expressed in (nucleoside-diphosphate kinase) |
| ZNF252 | 286101 | zinc finger protein 252 |
| FRS2 | 10818 | fibroblast growth factor receptor substrate 2 |
| SRR | 63826 | serine racemase |
| NPEPL1 | 79716 | aminopeptidase-like 1 |
| POLR3H | 171568 | polymerase (RNA) III (DNA directed) polypeptide H (22.9kD) |
| PHF8 | 23133 | PHD finger protein 8 |
| ZBTB39 | 9880 | zinc finger and BTB domain containing 39 |
| TRIP11 | 9321 | thyroid hormone receptor interactor 11 |
| PVR | 5817 | poliovirus receptor |
| POLR2J4 |  |  |
| C2orf43 | 60526 | chromosome 2 open reading frame 43 |
| RANBP3 | 8498 | RAN binding protein 3 |
| PDPR | 55066 | pyruvate dehydrogenase phosphatase regulatory subunit |
| ASH1L | 55870 | ash1 (absent, small, or homeotic)-like (Drosophila) |
| TMEM20 | 159371 | transmembrane protein 20 |
| ZCCHC4 | 29063 | zinc finger, CCHC domain containing 4 |
| C17orf63 | 55731 | chromosome 17 open reading frame 63 |
| FARP2 | 9855 | FERM, RhoGEF and pleckstrin domain protein 2 |
| LRRC40 | 55631 | leucine rich repeat containing 40 |
| ZNF783 | 155060 | zinc finger family member 783 |
| DVL2 | 1856 | dishevelled, dsh homolog 2 (Drosophila) |
| DCAKD | 79877 | dephospho-CoA kinase domain containing |
| FAM40A | 85369 | family with sequence similarity 40, member A |
| AKAP10 | 11216 | A kinase (PRKA) anchor protein 10 |
| UBE4B | 10277 | ubiquitination factor E4B (UFD2 homolog, yeast) |
| MYNN | 55892 | myoneurin |
| PTCD1 | 26024 | pentatricopeptide repeat domain 1 |
| DOPEY1 | 23033 | dopey family member 1 |
| RAB28 | 9364 | RAB28, member RAS oncogene family |
| OTUD7B | 56957 | OTU domain containing 7B |
| BCL6B | 255877 | B-cell CLL/lymphoma 6, member B (zinc finger protein) |
| TM9SF4 | 9777 | transmembrane 9 superfamily protein member 4 |
| ZNF254 | 9534 | zinc finger protein 254 |
| ESCO1 | 114799 | establishment of cohesion 1 homolog 1 (S. cerevisiae) |
| N6AMT1 | 29104 | N-6 adenine-specific DNA methyltransferase 1 (putative) |
| AKT2 | 208 | v-akt murine thymoma viral oncogene homolog 2 |
| C6orf162 | 57150 | chromosome 6 open reading frame 162 |
| SIRT3 | 23410 | sirtuin (silent mating type information regulation 2 homolog) 3 (S. cerevisiae) |
| AZI2 | 64343 | 5-azacytidine induced 2 |
| METT10D | 79066 | methyltransferase 10 domain containing |
| LCA5 | 167691 | Leber congenital amaurosis 5 |
| CHD6 | 84181 | chromodomain helicase DNA binding protein 6 |
| TRMT61A | 115708 | tRNA methyltransferase 61 homolog A (S. cerevisiae) |
| WHSC1L1 | 54904 | Wolf-Hirschhorn syndrome candidate 1-like 1 |
| VCPIP1 | 80124 | valosin containing protein (p97)/p47 complex interacting protein 1 |
| MAP3K7IP1 | 10454 | mitogen-activated protein kinase kinase kinase 7 interacting protein 1 |
| ELMOD2 | 255520 | ELMO/CED-12 domain containing 2 |
| DEDD | 9191 | death effector domain containing |
| ZNF223 | 7766 | zinc finger protein 223 |
| TMEM39A | 55254 | transmembrane protein 39A |
| C14orf118 | 55668 | chromosome 14 open reading frame 118 |
| ZSCAN29 | 146050 | zinc finger and SCAN domain containing 29 |
| FAM98C | 147965 | family with sequence similarity 98, member C |
| BNIP1 | 662 | BCL2/adenovirus E1B 19kDa interacting protein 1 |
| RBBP9 | 10741 | retinoblastoma binding protein 9 |
| ZNF48 | 197407 | zinc finger protein 48 |
| GEMIN8 | 54960 | gem (nuclear organelle) associated protein 8 |
| DISC1 | 27185 | disrupted in schizophrenia 1 |
| ZNF14 | 7561 | zinc finger protein 14 |
| BRF2 | 55290 | BRF2, subunit of RNA polymerase III transcription initiation factor, BRF1-like |
| CTPS2 | 56474 | CTP synthase II |
| SYMPK | 8189 | symplekin |
| NFAT5 | 10725 | nuclear factor of activated T-cells 5, tonicity-responsive |
| MTAP | 4507 | methylthioadenosine phosphorylase |
| MLLT6 | 4302 | myeloid/lymphoid or mixed-lineage leukemia (trithorax homolog, Drosophila); translocated to, 6 |
| AFTPH | 54812 | aftiphilin |
| USP40 | 55230 | ubiquitin specific peptidase 40 |
| ZNF579 | 84922 | zinc finger protein 579 |
| C1orf84 | 23334 | chromosome 1 open reading frame 84 |
| LRRC28 | 123355 | leucine rich repeat containing 28 |
| DNHD1 | 144132 | dynein heavy chain domain 1 |
| KIAA0841 | 23354 | KIAA0841 |
| ZFP41 | 2738 | zinc finger protein 41 homolog (mouse) |
| C1orf50 | 79078 | chromosome 1 open reading frame 50 |
| RAB30 | 27314 | RAB30, member RAS oncogene family |
| C16orf70 | 80262 | chromosome 16 open reading frame 70 |
| USP49 | 25862 | ubiquitin specific peptidase 49 |
| ZNF606 | 80095 | zinc finger protein 606 |
| OR1I1 | 126370 | olfactory receptor, family 1, subfamily I, member 1 |
| SLC24A1 | 9187 | solute carrier family 24 (sodium/potassium/calcium exchanger), member 1 |
| HPS1 | 3257 | Hermansky-Pudlak syndrome 1 |
| NKPD1 | 284353 | NTPase, KAP family P-loop domain containing 1 |
| BTRC | 8945 | beta-transducin repeat containing |
| L3MBTL | 26013 | l(3)mbt-like (Drosophila) |
| KIAA1731 | 85459 | KIAA1731 |
| TAF1 | 1863 | TAF1 RNA polymerase II, TATA box binding protein (TBP)-associated factor, 250kDa |
| POLR2C | 5432 | polymerase (RNA) II (DNA directed) polypeptide C, 33kDa |
| KIAA0586 | 9786 | KIAA0586 |
| FICD | 11153 | FIC domain containing |
| SLC46A1 | 113235 | solute carrier family 46 (folate transporter), member 1 |
| B4GALT7 | 11285 | xylosylprotein beta 1,4-galactosyltransferase, polypeptide 7 (galactosyltransferase I) |
| CCDC40 | 55036 | coiled-coil domain containing 40 |
| MESP2 | 145873 | mesoderm posterior 2 homolog (mouse) |
| CCDC132 | 55610 | coiled-coil domain containing 132 |
| PI4KB | 5298 | phosphatidylinositol 4-kinase, catalytic, beta |
| RRP1 | 8568 | ribosomal RNA processing 1 homolog (S. cerevisiae) |
| NSD1 | 64324 | nuclear receptor binding SET domain protein 1 |
| CNOT4 | 4850 | CCR4-NOT transcription complex, subunit 4 |
| C22orf30 | 253143 | chromosome 22 open reading frame 30 |
| C3orf19 | 51244 | chromosome 3 open reading frame 19 |
| KCNJ14 | 3770 | potassium inwardly-rectifying channel, subfamily J, member 14 |
| WDR31 | 114987 | WD repeat domain 31 |
| WDR35 | 57539 | WD repeat domain 35 |
| AKAP8 | 10270 | A kinase (PRKA) anchor protein 8 |
| TK2 | 7084 | thymidine kinase 2, mitochondrial |
| RBMX2 | 51634 | RNA binding motif protein, X-linked 2 |
| ZNF248 | 57209 | zinc finger protein 248 |
| CRCP | 27297 | CGRP receptor component |
| RPAP2 | 79871 | RNA polymerase II associated protein 2 |
| TIGD7 | 91151 | tigger transposable element derived 7 |
| C22orf40 | 150383 | chromosome 22 open reading frame 40 |
| FAM173B | 134145 | family with sequence similarity 173, member B |
| ZNF85 | 7639 | zinc finger protein 85 |
| FRS3 | 10817 | fibroblast growth factor receptor substrate 3 |
| ZNF609 | 23060 | zinc finger protein 609 |
| SNX13 | 23161 | sorting nexin 13 |
| ZNF720 | 124411 | zinc finger protein 720 |
| ZNF16 | 7564 | zinc finger protein 16 |
| MFSD8 | 256471 | major facilitator superfamily domain containing 8 |
| C12orf72 | 254013 | chromosome 12 open reading frame 72 |
| C5orf51 | 285636 | chromosome 5 open reading frame 51 |
| ZMYM5 | 9205 | zinc finger, MYM-type 5 |
| ZNF778 | 197320 | zinc finger protein 778 |
| NUDT17 | 200035 | nudix (nucleoside diphosphate linked moiety X)-type motif 17 |
| LZTS2 | 84445 | leucine zipper, putative tumor suppressor 2 |
| USP19 | 10869 | ubiquitin specific peptidase 19 |
| C15orf29 | 79768 | chromosome 15 open reading frame 29 |
| MLLT1 | 4298 | myeloid/lymphoid or mixed-lineage leukemia (trithorax homolog, Drosophila); translocated to, 1 |
| ZNF780B | 163131 | zinc finger protein 780B |
| GOSR2 | 9570 | golgi SNAP receptor complex member 2 |
| MCPH1 | 79648 | microcephalin 1 |
| C8orf30A | 51236 | chromosome 8 open reading frame 30A |
| BAZ2A | 11176 | bromodomain adjacent to zinc finger domain, 2A |
| LOC728198 | 51616 | similar to transcription associated factor TAFII31L |
| C7orf38 | 221786 | chromosome 7 open reading frame 38 |
| C3orf31 | 132001 | chromosome 3 open reading frame 31 |
| ATF6 | 22926 | activating transcription factor 6 |
| AMZ1 | 155185 | archaelysin family metallopeptidase 1 |
| ZNF449 | 203523 | zinc finger protein 449 |
| ATG2B | 55102 | ATG2 autophagy related 2 homolog B (S. cerevisiae) |
| PATZ1 | 23598 | POZ (BTB) and AT hook containing zinc finger 1 |
| ZNF419 | 79744 | zinc finger protein 419 |
| SEC22C | 9117 | SEC22 vesicle trafficking protein homolog C (S. cerevisiae) |
| TIRAP | 114609 | toll-interleukin 1 receptor (TIR) domain containing adaptor protein |
| L3MBTL2 | 83746 | l(3)mbt-like 2 (Drosophila) |
| VPS8 | 23355 | vacuolar protein sorting 8 homolog (S. cerevisiae) |
| AMBRA1 | 55626 | autophagy/beclin-1 regulator 1 |
| ZNF709 | 163050 | zinc finger protein 709 |
| ZNF93 | 81931 | zinc finger protein 93 |
| COG6 | 57511 | component of oligomeric golgi complex 6 |
| NF2 | 4771 | neurofibromin 2 (merlin) |
| SDAD1 | 55153 | SDA1 domain containing 1 |
| PLD2 | 5338 | phospholipase D2 |
| ZSWIM1 | 90204 | zinc finger, SWIM-type containing 1 |
| CHCHD5 | 84269 | coiled-coil-helix-coiled-coil-helix domain containing 5 |
| ZBTB17 | 7709 | zinc finger and BTB domain containing 17 |
| CCDC9 | 26093 | coiled-coil domain containing 9 |
| MAP2K7 | 5609 | mitogen-activated protein kinase kinase 7 |
| RPL32P3 | 132241 | ribosomal protein L32 pseudogene 3 |
| LRIG2 | 9860 | leucine-rich repeats and immunoglobulin-like domains 2 |
| KIAA1704 | 55425 | KIAA1704 |
| NCAPH2 | 29781 | non-SMC condensin II complex, subunit H2 |
| LOC400236 |  |  |
| KLHL22 | 84861 | kelch-like 22 (Drosophila) |
| SMUG1 | 23583 | single-strand-selective monofunctional uracil-DNA glycosylase 1 |
| GATAD2B | 57459 | GATA zinc finger domain containing 2B |
| ZFYVE20 | 64145 | zinc finger, FYVE domain containing 20 |
| GPR107 | 57720 | G protein-coupled receptor 107 |
| ZNF2 | 7549 | zinc finger protein 2 |
| LOC80054 |  |  |
| FOXK1 | 221937 | forkhead box K1 |
| ZNF669 | 79862 | zinc finger protein 669 |
| DMWD | 1762 | dystrophia myotonica, WD repeat containing |
| MAP3K13 | 9175 | mitogen-activated protein kinase kinase kinase 13 |
| ABCB8 | 11194 | ATP-binding cassette, sub-family B (MDR/TAP), member 8 |
| TCEANC | 170082 | transcription elongation factor A (SII) N-terminal and central domain containing |
| ARMC7 | 79637 | armadillo repeat containing 7 |
| DNAJC4 | 3338 | DnaJ (Hsp40) homolog, subfamily C, member 4 |
| TSEN2 | 80746 | tRNA splicing endonuclease 2 homolog (S. cerevisiae) |
| ZNF354C | 30832 | zinc finger protein 354C |
| GOLGA1 | 2800 | golgi autoantigen, golgin subfamily a, 1 |
| C21orf2 | 755 | chromosome 21 open reading frame 2 |
| HDAC6 | 10013 | histone deacetylase 6 |
| ACAD10 | 80724 | acyl-Coenzyme A dehydrogenase family, member 10 |
| METT5D1 | 196074 | methyltransferase 5 domain containing 1 |
| PRKRIP1 | 79706 | PRKR interacting protein 1 (IL11 inducible) |
| PBX2 | 5089 | pre-B-cell leukemia homeobox 2 |
| C12orf30 | 80018 | chromosome 12 open reading frame 30 |
| RFX1 | 5989 | regulatory factor X, 1 (influences HLA class II expression) |
| C4orf29 | 80167 | chromosome 4 open reading frame 29 |
| POMT2 | 29954 | protein-O-mannosyltransferase 2 |
| CENPP | 401541 | centromere protein P |
| JARID1C | 8242 | jumonji, AT rich interactive domain 1C |
| SFRS8 | 6433 | splicing factor, arginine/serine-rich 8 (suppressor-of-white-apricot homolog, Drosophila) |
| C20orf29 | 55317 | chromosome 20 open reading frame 29 |
| ABCF3 | 55324 | ATP-binding cassette, sub-family F (GCN20), member 3 |
| ORC5L | 5001 | origin recognition complex, subunit 5-like (yeast) |
| UBOX5 | 22888 | U-box domain containing 5 |
| HDGF2 | 84717 | hepatoma-derived growth factor-related protein 2 |
| ADCK4 | 79934 | aarF domain containing kinase 4 |
| ATXN3 | 4287 | ataxin 3 |
| C1orf91 | 56063 | chromosome 1 open reading frame 91 |
| ZNF174 | 7727 | zinc finger protein 174 |
| SAPS2 | 9701 | SAPS domain family, member 2 |
| STAM2 | 10254 | signal transducing adaptor molecule (SH3 domain and ITAM motif) 2 |
| FLCN | 201163 | folliculin |
| FBXO42 | 54455 | F-box protein 42 |
| SUFU | 51684 | suppressor of fused homolog (Drosophila) |
| ZNF362 | 149076 | zinc finger protein 362 |
| SNAP47 | 116841 | synaptosomal-associated protein, 47kDa |
| NEK11 | 79858 | NIMA (never in mitosis gene a)- related kinase 11 |
| SEPSECS | 51091 | Sep (O-phosphoserine) tRNA:Sec (selenocysteine) tRNA synthase |
| UBE2CBP | 90025 | ubiquitin-conjugating enzyme E2C binding protein |
| NMNAT1 | 64802 | nicotinamide nucleotide adenylyltransferase 1 |
| DNAJB12 | 54788 | DnaJ (Hsp40) homolog, subfamily B, member 12 |
| TRIM3 | 10612 | tripartite motif-containing 3 |
| ZNF213 | 7760 | zinc finger protein 213 |
| DNASE2 | 1777 | deoxyribonuclease II, lysosomal |
| GBF1 | 8729 | golgi-specific brefeldin A resistant guanine nucleotide exchange factor 1 |
| MBD5 | 55777 | methyl-CpG binding domain protein 5 |
| ELAC1 | 55520 | elaC homolog 1 (E. coli) |
| EEPD1 | 80820 | endonuclease/exonuclease/phosphatase family domain containing 1 |
| SAMD4B | 55095 | sterile alpha motif domain containing 4B |
| ZSCAN20 | 7579 | zinc finger and SCAN domain containing 20 |
| XPNPEP3 | 63929 | X-prolyl aminopeptidase (aminopeptidase P) 3, putative |
| C2orf18 | 54978 | chromosome 2 open reading frame 18 |
| DNAH7 | 56171 | dynein, axonemal, heavy chain 7 |
| ZNF384 | 171017 | zinc finger protein 384 |
| NF1 | 4763 | neurofibromin 1 |
| GPR156 | 165829 | G protein-coupled receptor 156 |
| KIAA0141 | 9812 | KIAA0141 |
| RAD52 | 5893 | RAD52 homolog (S. cerevisiae) |
| ZNF260 | 339324 | zinc finger protein 260 |
| PGBD3 | 2074 | piggyBac transposable element derived 3 |
| KIAA0894 | 22833 | KIAA0894 protein |
| TRAFD1 | 10906 | TRAF-type zinc finger domain containing 1 |
| TNPO3 | 23534 | transportin 3 |
| PURB | 5814 | purine-rich element binding protein B |
| USP37 | 57695 | ubiquitin specific peptidase 37 |
| C19orf40 | 91442 | chromosome 19 open reading frame 40 |
| RABL3 | 285282 | RAB, member of RAS oncogene family-like 3 |
| PTAR1 | 375743 | protein prenyltransferase alpha subunit repeat containing 1 |
| ZBTB33 | 10009 | zinc finger and BTB domain containing 33 |
| C10orf12 | 26148 | chromosome 10 open reading frame 12 |
| UBAP1 | 51271 | ubiquitin associated protein 1 |
| CDK5RAP2 | 55755 | CDK5 regulatory subunit associated protein 2 |
| SLC39A9 | 55334 | solute carrier family 39 (zinc transporter), member 9 |
| ZCCHC3 | 85364 | zinc finger, CCHC domain containing 3 |
| AP4S1 | 11154 | adaptor-related protein complex 4, sigma 1 subunit |
| ZNF576 | 79177 | zinc finger protein 576 |
| PHKA2 | 5256 | phosphorylase kinase, alpha 2 (liver) |
| SEMA4F | 10505 | sema domain, immunoglobulin domain (Ig), transmembrane domain (TM) and short cytoplasmic domain, (semaphorin) 4F |
| IRGQ | 126298 | immunity-related GTPase family, Q |
| HIRIP3 | 8479 | HIRA interacting protein 3 |
| C10orf88 | 80007 | chromosome 10 open reading frame 88 |
| HDAC8 | 55869 | histone deacetylase 8 |
| LIMD1 | 8994 | LIM domains containing 1 |
| SLC22A25 | 387601 | solute carrier family 22, member 25 |
| INO80D | 54891 | INO80 complex subunit D |
| DHRS12 | 79758 | dehydrogenase/reductase (SDR family) member 12 |
| ZNF35 | 7584 | zinc finger protein 35 |
| ZNF445 | 353274 | zinc finger protein 445 |
| TTC23 | 64927 | tetratricopeptide repeat domain 23 |
| C20orf200 | 253868 | chromosome 20 open reading frame 200 |
| MID2 | 11043 | midline 2 |
| BOLA1 | 51027 | bolA homolog 1 (E. coli) |
| RNF170 | 81790 | ring finger protein 170 |
| GALK2 | 2585 | galactokinase 2 |
| FUK | 197258 | fucokinase |
| UBE2J2 | 118424 | ubiquitin-conjugating enzyme E2, J2 (UBC6 homolog, yeast) |
| TCEB3 | 6924 | transcription elongation factor B (SIII), polypeptide 3 (110kDa, elongin A) |
| KPNA6 | 23633 | karyopherin alpha 6 (importin alpha 7) |
| C1orf69 | 200205 | chromosome 1 open reading frame 69 |
| VPS53 | 55275 | vacuolar protein sorting 53 homolog (S. cerevisiae) |
| MSH3 | 4437 | mutS homolog 3 (E. coli) |
| ZNF333 | 84449 | zinc finger protein 333 |
| BRWD3 | 254065 | bromodomain and WD repeat domain containing 3 |
| RNASEH2B | 79621 | ribonuclease H2, subunit B |
| ZFP64 | 55734 | zinc finger protein 64 homolog (mouse) |
| FANCC | 2176 | Fanconi anemia, complementation group C |
| ERC1 | 23085 | ELKS/RAB6-interacting/CAST family member 1 |
| C2orf60 | 129450 | chromosome 2 open reading frame 60 |
| C5orf37 | 134359 | chromosome 5 open reading frame 37 |
| C6orf89 | 221477 | chromosome 6 open reading frame 89 |
| UGCGL1 | 56886 | UDP-glucose ceramide glucosyltransferase-like 1 |
| ZNF253 | 56242 | zinc finger protein 253 |
| C17orf56 | 146705 | chromosome 17 open reading frame 56 |
| RAD51L3 | 5892 | RAD51-like 3 (S. cerevisiae) |
| ZNF498 | 221785 | zinc finger protein 498 |
| ZNF770 | 54989 | zinc finger protein 770 |
| C17orf69 | 147081 | chromosome 17 open reading frame 69 |
| GSK3A | 2931 | glycogen synthase kinase 3 alpha |
| ZNF226 | 7769 | zinc finger protein 226 |
| TMEM62 | 80021 | transmembrane protein 62 |
| CCDC25 | 55246 | coiled-coil domain containing 25 |
| ZNF585A | 199704 | zinc finger protein 585A |
| SLC38A7 | 55238 | solute carrier family 38, member 7 |
| IKZF5 | 64376 | IKAROS family zinc finger 5 (Pegasus) |
| NFX1 | 4799 | nuclear transcription factor, X-box binding 1 |
| AARSD1 | 80755 | alanyl-tRNA synthetase domain containing 1 |
| C19orf29 | 58509 | chromosome 19 open reading frame 29 |
| NACC1 | 112939 | nucleus accumbens associated 1, BEN and BTB (POZ) domain containing |
| NCRNA00085 | 27113 | non-protein coding RNA 85 |
| ZNF688 | 146540 | zinc finger protein 688 |
| RGS12 | 6002 | regulator of G-protein signaling 12 |
| RP5-1000E10.4 | 80143 | suppressor of IKK epsilon |
| ZNF398 | 57541 | zinc finger protein 398 |
| VPS52 | 6293 | vacuolar protein sorting 52 homolog (S. cerevisiae) |
| TRIM66 | 9866 | tripartite motif-containing 66 |
| IVD | 3712 | isovaleryl Coenzyme A dehydrogenase |
| CCDC123 | 84902 | coiled-coil domain containing 123 |
| C16orf13 | 84326 | chromosome 16 open reading frame 13 |
| C7orf67 | 84182 | chromosome 7 open reading frame 67 |
| LOC727778 | 56900 | similar to protein x 013 |
| CRKRS | 51755 | Cdc2-related kinase, arginine/serine-rich |
| C6orf35 | 729515 | chromosome 6 open reading frame 35 |
| TBRG1 | 84897 | transforming growth factor beta regulator 1 |
| GPATCH2 | 55105 | G patch domain containing 2 |
| CIR | 9541 | CBF1 interacting corepressor |
| CALCOCO1 | 57658 | calcium binding and coiled-coil domain 1 |
| C18orf22 | 79863 | chromosome 18 open reading frame 22 |
| TRAF3IP1 | 26146 | TNF receptor-associated factor 3 interacting protein 1 |
| RABL4 | 11020 | RAB, member of RAS oncogene family-like 4 |
| POLR3B | 55703 | polymerase (RNA) III (DNA directed) polypeptide B |
| ZKSCAN5 | 23660 | zinc finger with KRAB and SCAN domains 5 |
| ZNF507 | 22847 | zinc finger protein 507 |
| FAM172A | 83989 | family with sequence similarity 172, member A |
| RSPH3 | 83861 | radial spoke 3 homolog (Chlamydomonas) |
| ERCC8 | 1161 | excision repair cross-complementing rodent repair deficiency, complementation group 8 |
| UBN2 | 254048 | ubinuclein 2 |
| TRIM39 | 56658 | tripartite motif-containing 39 |
| JRK |  |  |
| NRF1 | 4899 | nuclear respiratory factor 1 |
| MYST1 | 84148 | MYST histone acetyltransferase 1 |
| C2orf49 | 79074 | chromosome 2 open reading frame 49 |
| KLHL20 | 27252 | kelch-like 20 (Drosophila) |
| ACVR2A | 92 | activin A receptor, type IIA |
| TLK2 | 11011 | tousled-like kinase 2 |
| FBXW9 | 84261 | F-box and WD repeat domain containing 9 |
| LZTR1 | 8216 | leucine-zipper-like transcription regulator 1 |
| ZNF197 | 10168 | zinc finger protein 197 |
| ANGEL1 | 23357 | angel homolog 1 (Drosophila) |
| DNASE1 | 1773 | deoxyribonuclease I |
| KIAA0556 | 23247 | KIAA0556 |
| C11orf30 | 56946 | chromosome 11 open reading frame 30 |
| ASB1 | 51665 | ankyrin repeat and SOCS box-containing 1 |
| GTDC1 | 79712 | glycosyltransferase-like domain containing 1 |
| MKS1 | 54903 | Meckel syndrome, type 1 |
| ZNF496 | 84838 | zinc finger protein 496 |
| TRUB1 | 142940 | TruB pseudouridine (psi) synthase homolog 1 (E. coli) |
| DSTYK | 25778 | dual serine/threonine and tyrosine protein kinase |
| RNF121 | 55298 | ring finger protein 121 |
| C9orf85 | 138241 | chromosome 9 open reading frame 85 |
| CCDC33 | 80125 | coiled-coil domain containing 33 |
| SETDB1 | 9869 | SET domain, bifurcated 1 |
| DTX2 | 113878 | deltex homolog 2 (Drosophila) |
| CCDC93 | 54520 | coiled-coil domain containing 93 |
| RECQL5 | 9400 | RecQ protein-like 5 |
| LUZP1 | 7798 | leucine zipper protein 1 |
| HLCS | 3141 | holocarboxylase synthetase (biotin-(proprionyl-Coenzyme A-carboxylase (ATP-hydrolysing)) ligase) |
| HS1BP3 | 64342 | HCLS1 binding protein 3 |
| FAM3A | 60343 | family with sequence similarity 3, member A |
| XIAP | 331 | X-linked inhibitor of apoptosis |
| PYGO2 | 90780 | pygopus homolog 2 (Drosophila) |
| C2CD3 | 26005 | C2 calcium-dependent domain containing 3 |
| AP4E1 | 23431 | adaptor-related protein complex 4, epsilon 1 subunit |
| FAF2 | 23197 | Fas associated factor family member 2 |
| MAP3K2 | 10746 | mitogen-activated protein kinase kinase kinase 2 |
| TUBGCP5 | 114791 | tubulin, gamma complex associated protein 5 |
| C14orf43 | 91748 | chromosome 14 open reading frame 43 |
| IPO8 | 10526 | importin 8 |
| SNAPC3 | 6619 | small nuclear RNA activating complex, polypeptide 3, 50kDa |
| PACS1 | 55690 | phosphofurin acidic cluster sorting protein 1 |
| RNGTT | 8732 | RNA guanylyltransferase and 5'-phosphatase |
| IKZF4 | 64375 | IKAROS family zinc finger 4 (Eos) |
| FAM135B | 51059 | family with sequence similarity 135, member B |
| DIS3 | 22894 | DIS3 mitotic control homolog (S. cerevisiae) |
| BBS9 | 27241 | Bardet-Biedl syndrome 9 |
| MAN1A2 | 10905 | mannosidase, alpha, class 1A, member 2 |
| HNRNPUL2 | 221092 | heterogeneous nuclear ribonucleoprotein U-like 2 |
| RBM33 | 155435 | RNA binding motif protein 33 |
| TCHP | 84260 | trichoplein, keratin filament binding |
| JRKL | 8690 | jerky homolog-like (mouse) |
| ZNF502 | 91392 | zinc finger protein 502 |
| EPB41L4A | 64097 | erythrocyte membrane protein band 4.1 like 4A |
| C15orf40 | 123207 | chromosome 15 open reading frame 40 |
| PTPN9 | 5780 | protein tyrosine phosphatase, non-receptor type 9 |
| FUT10 | 84750 | fucosyltransferase 10 (alpha (1,3) fucosyltransferase) |
| C10orf84 | 63877 | chromosome 10 open reading frame 84 |
| C7orf36 | 57002 | chromosome 7 open reading frame 36 |
| C12orf47 |  | chromosome 12open reading frame 47 |
| LOC646808 |  |  |
| CDADC1 | 81602 | cytidine and dCMP deaminase domain containing 1 |
| WIZ | 58525 | widely interspaced zinc finger motifs |
| C19orf25 | 148223 | chromosome 19 open reading frame 25 |
| WDTC1 | 23038 | WD and tetratricopeptide repeats 1 |
| PPP2R5D | 5528 | protein phosphatase 2, regulatory subunit B', delta isoform |
| PEX16 | 9409 | peroxisomal biogenesis factor 16 |
| SIRT6 | 51548 | sirtuin (silent mating type information regulation 2 homolog) 6 (S. cerevisiae) |
| ERCC4 | 2072 | excision repair cross-complementing rodent repair deficiency, complementation group 4 |
| SEC22A | 26984 | SEC22 vesicle trafficking protein homolog A (S. cerevisiae) |
| MVK | 4598 | mevalonate kinase |
| DDX6 | 1656 | DEAD (Asp-Glu-Ala-Asp) box polypeptide 6 |
| SLC35C2 | 51006 | solute carrier family 35, member C2 |
| LARP2 | 55132 | La ribonucleoprotein domain family, member 2 |
| STX17 | 55014 | syntaxin 17 |
| ZNF839 | 55778 | zinc finger protein 839 |
| ANAPC7 | 51434 | anaphase promoting complex subunit 7 |
| DGCR14 | 8220 | DiGeorge syndrome critical region gene 14 |
| KLHL18 | 23276 | kelch-like 18 (Drosophila) |
| ENOX2 | 10495 | ecto-NOX disulfide-thiol exchanger 2 |
| CIAO1 | 9391 | cytosolic iron-sulfur protein assembly 1 homolog (S. cerevisiae) |
| ZNF529 | 57711 | zinc finger protein 529 |
| ZNF34 | 80778 | zinc finger protein 34 |
| TSPAN31 | 6302 | tetraspanin 31 |
| LMLN | 89782 | leishmanolysin-like (metallopeptidase M8 family) |
| PAFAH1B2 | 5049 | platelet-activating factor acetylhydrolase, isoform Ib, beta subunit 30kDa |
| RTEL1 | 8771 | regulator of telomere elongation helicase 1 |
| KIAA0652 | 9776 | KIAA0652 |
| ZNF396 | 252884 | zinc finger protein 396 |
| API5 | 8539 | apoptosis inhibitor 5 |
| C3orf17 | 25871 | chromosome 3 open reading frame 17 |
| TAOK2 | 9344 | TAO kinase 2 |
| RPRD2 | 23248 | regulation of nuclear pre-mRNA domain containing 2 |
| MKLN1 | 4289 | muskelin 1, intracellular mediator containing kelch motifs |
| WHAMML1 | 123720 | WAS protein homolog associated with actin, golgi membranes and microtubules-like 1 |
| WIBG | 84305 | within bgcn homolog (Drosophila) |
| UBE2W | 55284 | ubiquitin-conjugating enzyme E2W (putative) |
| TYW1 | 55253 | tRNA-yW synthesizing protein 1 homolog (S. cerevisiae) |
| EARS2 | 124454 | glutamyl-tRNA synthetase 2, mitochondrial (putative) |
| IQCH | 64799 | IQ motif containing H |
| SMU1 | 55234 | smu-1 suppressor of mec-8 and unc-52 homolog (C. elegans) |
| MIER2 | 54531 | mesoderm induction early response 1, family member 2 |
| NUFIP2 | 57532 | nuclear fragile X mental retardation protein interacting protein 2 |
| CXorf39 | 139231 | chromosome X open reading frame 39 |
| USP30 | 84749 | ubiquitin specific peptidase 30 |
| BLOC1S3 | 388552 | biogenesis of lysosomal organelles complex-1, subunit 3 |
| ZNF641 | 121274 | zinc finger protein 641 |
| TAF6L | 10629 | TAF6-like RNA polymerase II, p300/CBP-associated factor (PCAF)-associated factor, 65kDa |
| ZNF594 | 84622 | zinc finger protein 594 |
| C6orf182 | 285753 | chromosome 6 open reading frame 182 |
| DNAH1 | 25981 | dynein, axonemal, heavy chain 1 |
| MLL2 | 8085 | myeloid/lymphoid or mixed-lineage leukemia 2 |
| EYA3 | 2140 | eyes absent homolog 3 (Drosophila) |
| N4BP1 | 9683 | NEDD4 binding protein 1 |
| CRAMP1L | 57585 | Crm, cramped-like (Drosophila) |
| SCLY | 51540 | selenocysteine lyase |
| FAM98B | 283742 | family with sequence similarity 98, member B |
| INTS4 | 92105 | integrator complex subunit 4 |
| IGHMBP2 | 3508 | immunoglobulin mu binding protein 2 |
| METTL10 | 399818 | methyltransferase like 10 |
| RSPRY1 | 89970 | ring finger and SPRY domain containing 1 |
| AP3S2 | 10239 | adaptor-related protein complex 3, sigma 2 subunit |
| THAP6 | 152815 | THAP domain containing 6 |
| ST20 | 400410 | suppressor of tumorigenicity 20 |
| C17orf57 | 124989 | chromosome 17 open reading frame 57 |
| ZNF236 | 7776 | zinc finger protein 236 |
| LRRC58 | 116064 | leucine rich repeat containing 58 |
| CNNM2 | 54805 | cyclin M2 |
| ENSG00000006837 |  | Cyclin-dependent kinase-like 3 (EC 2.7.11.22)(Serine/threonine protein kinase NKIAMRE) [Source:UniProtKB/Swiss-Prot;Acc:Q8IVW4] |
| MPV17L | 255027 | MPV17 mitochondrial membrane protein-like |
| SUPT6H | 6830 | suppressor of Ty 6 homolog (S. cerevisiae) |
| SENP5 | 205564 | SUMO1/sentrin specific peptidase 5 |
| LOC222070 |  |  |
| C1orf35 | 79169 | chromosome 1 open reading frame 35 |
| SERGEF | 26297 | secretion regulating guanine nucleotide exchange factor |
| CLUAP1 | 23059 | clusterin associated protein 1 |

SI dataset 4. CD4+ T cell specific expressed miRNA.

hsa-let-7f-1,hsa-let-7f-2,5p

hsa-let-7d,5p

hsa-let-7g,5p

hsa-let-7i,5p

hsa-mir-652,3p

hsa-mir-101-1,hsa-mir-101-2,3p

hsa-mir-17,5p

hsa-mir-20a,5p

hsa-mir-20b,5p

hsa-mir-106b,5p

hsa-mir-139,3p

hsa-mir-140,3p

hsa-mir-142,3p

hsa-mir-142,5p

hsa-mir-146a,5p

hsa-mir-150,5p

hsa-mir-155,5p

hsa-mir-15a,5p

hsa-mir-15b,5p

hsa-mir-181a-1,hsa-mir-181a-2,5p

hsa-mir-181b-1,hsa-mir-181b-2,5p

hsa-mir-182,5p

hsa-mir-18a,5p

hsa-mir-19b-1,hsa-mir-19b-2,3p

hsa-mir-19a,3p

hsa-mir-210,3p

hsa-mir-223,3p

hsa-mir-92a-1,hsa-mir-92a-2,3p

hsa-mir-26a-2,5p

hsa-mir-29b-1,hsa-mir-29b-2,3p

hsa-mir-29a,3p

hsa-mir-301b,3p

hsa-mir-30e,5p

hsa-mir-32,5p

hsa-mir-33a,5p

hsa-mir-342,3p

hsa-mir-363,3p

hsa-mir-423,5p

hsa-mir-7-1,hsa-mir-7-2,hsa-mir-7-3,5p

hsa-mir-93,5p

hsa-mir-96,5p

SI dataset 5. Housekeeping miRNAs.

hsa-let-7a-1,hsa-let-7a-2,hsa-let-7a-3,hsa-let-7b,hsa-let-7c,5p

hsa-let-7a-1,hsa-let-7a-2,hsa-let-7a-3,hsa-let-7c,5p

hsa-let-7a-1,hsa-let-7a-2,hsa-let-7a-3,hsa-let-7c,hsa-mir-98,5p

hsa-let-7a-1,hsa-let-7a-2,hsa-let-7a-3,hsa-let-7d,5p

hsa-let-7a-1,hsa-let-7a-2,hsa-let-7a-3,hsa-let-7e,hsa-let-7f-1,5p

hsa-let-7a-1,hsa-let-7a-2,hsa-let-7a-3,hsa-let-7f-1,hsa-let-7f-2,5p

hsa-let-7a-1,hsa-let-7a-2,hsa-let-7a-3,hsa-let-7f-1,hsa-let-7f-2,hsa-let-7g,5p

hsa-let-7a-1,hsa-let-7a-2,hsa-let-7a-3,hsa-let-7g,5p

hsa-let-7a-1,hsa-let-7a-2,hsa-let-7a-3,hsa-mir-98,5p

hsa-let-7a-1,hsa-let-7c,5p

hsa-let-7a-1,hsa-let-7e,5p

hsa-let-7a-2,hsa-let-7c,5p

hsa-let-7a-2,hsa-let-7e,5p

hsa-let-7a-2,hsa-let-7f-1,hsa-let-7f-2,5p

hsa-let-7a-2,hsa-let-7f-2,5p

hsa-let-7c,hsa-mir-98,5p

hsa-let-7d,hsa-let-7f-1,hsa-let-7f-2,5p

hsa-let-7e,hsa-let-7f-1,hsa-let-7f-2,5p

hsa-let-7f-1,hsa-let-7f-2,hsa-let-7g,5p

hsa-let-7g,hsa-let-7i,5p

hsa-let-7a-3,5p

hsa-let-7f-1,5p

hsa-mir-1-2,3p

hsa-mir-672__FM,5p

hsa-mir-871__FM,5p

hsa-mir-532,3p

hsa-mir-532,5p

hsa-mir-615,3p

hsa-mir-615,5p

hsa-mir-872__FM,5p

hsa-mir-674__FR,3p

hsa-mir-674__FR,5p

hsa-mir-885,3p

hsa-mir-885,5p

hsa-mir-590,3p

hsa-mir-590,5p

hsa-mir-671,3p

hsa-mir-671,5p

hsa-mir-570,3p

hsa-mir-760,3p

hsa-mir-887,3p

hsa-mir-874,3p

hsa-mir-574,5p

hsa-mir-654,3p

hsa-mir-654,5p

hsa-mir-100871,3p

hsa-mir-551b,3p

hsa-mir-875,3p

hsa-mir-875,5p

hsa-mir-876,3p

hsa-mir-876,5p

hsa-mir-100,hsa-mir-99a,5p

hsa-mir-101-1,3p

hsa-mir-101-2,3p

hsa-mir-665,3p

hsa-mir-103-1,hsa-mir-103-2,hsa-mir-107,3p

hsa-mir-107,3p

hsa-mir-105-1,hsa-mir-105-2,5p

hsa-mir-106a,hsa-mir-17,hsa-mir-20a,5p

hsa-mir-106a,hsa-mir-17,hsa-mir-20b,5p

hsa-mir-17,hsa-mir-20a,5p

hsa-mir-17,hsa-mir-20a,hsa-mir-20b,5p

hsa-mir-17,hsa-mir-20b,5p

hsa-mir-20a,hsa-mir-20b,5p

hsa-mir-758,3p

hsa-mir-208b,3p

hsa-mir-889,3p

hsa-mir-124-1,3p

hsa-mir-125b-1,5p

hsa-mir-125b-2,5p

hsa-mir-127,3p

hsa-mir-127,5p

hsa-mir-130a,hsa-mir-130b,3p

hsa-mir-133a-1,hsa-mir-133a-2,3p

hsa-mir-133a-1,hsa-mir-133a-2,hsa-mir-133b,3p

hsa-mir-138-1,5p

hsa-mir-138-2,5p

hsa-mir-139,3p

hsa-mir-141,hsa-mir-200a,3p

hsa-mir-146a,hsa-mir-146b,5p

hsa-mir-146b,3p

hsa-mir-146b,5p

hsa-mir-147b,3p

hsa-mir-149,5p

hsa-mir-15a,hsa-mir-15b,5p

hsa-mir-16-1,hsa-mir-16-2,hsa-mir-195,5p

hsa-mir-181a-1,hsa-mir-181a-2,hsa-mir-181c,5p

hsa-mir-181c,5p

hsa-mir-181b-1,hsa-mir-181b-2,hsa-mir-181d,5p

hsa-mir-181b-2,hsa-mir-181d,5p

hsa-mir-181b-1,5p

hsa-mir-181b-2,5p

hsa-mir-181d,5p

hsa-mir-184,3p

hsa-mir-187,3p

hsa-mir-188,3p

hsa-mir-188,5p

hsa-mir-18a,hsa-mir-18b,5p

hsa-mir-190,5p

hsa-mir-192,hsa-mir-215,5p

hsa-mir-215,5p

hsa-mir-196a-2,hsa-mir-196b,5p

hsa-mir-198,5p

hsa-mir-199a-1,hsa-mir-199a-2,hsa-mir-199b,5p

hsa-mir-199a-2,3p

hsa-mir-19a,hsa-mir-19b-1,3p

hsa-mir-19a,hsa-mir-19b-1,hsa-mir-19b-2,3p

hsa-mir-19a,hsa-mir-19b-2,3p

hsa-mir-19b-1,3p

hsa-mir-19b-2,3p

hsa-mir-655,3p

hsa-mir-891b,5p

hsa-mir-660,5p

hsa-mir-200b,hsa-mir-200c,3p

hsa-mir-206,3p

hsa-mir-216a,hsa-mir-216b,5p

hsa-mir-216b,5p

hsa-mir-218-1,5p

hsa-mir-219-1,hsa-mir-219-2,5p

hsa-mir-219-1,3p

hsa-mir-219-1,5p

hsa-mir-219-2,3p

hsa-mir-219-2,5p

hsa-mir-24-1,3p

hsa-mir-25,hsa-mir-92a-1,hsa-mir-92a-2,3p

hsa-mir-92a-1,hsa-mir-92a-2,hsa-mir-92b,3p

hsa-mir-92a-2,3p

hsa-mir-26a-1,hsa-mir-26a-2,hsa-mir-26b,5p

hsa-mir-26a-2,hsa-mir-26b,5p

hsa-mir-26a-1,5p

hsa-mir-296,3p

hsa-mir-296,5p

hsa-mir-299,3p

hsa-mir-299,5p

hsa-mir-29a,hsa-mir-29b-1,hsa-mir-29b-2,3p

hsa-mir-29a,hsa-mir-29c,3p

hsa-mir-29b-1,hsa-mir-29b-2,hsa-mir-29c,3p

hsa-mir-29b-1,3p

hsa-mir-29b-2,3p

hsa-mir-301a,hsa-mir-301b,3p

hsa-mir-302a,hsa-mir-302b,3p

hsa-mir-302a,hsa-mir-302b,hsa-mir-302c,hsa-mir-302d,3p

hsa-mir-302a,hsa-mir-302c,3p

hsa-mir-302a,hsa-mir-302d,3p

hsa-mir-302b,hsa-mir-302c,3p

hsa-mir-302c,hsa-mir-302d,3p

hsa-mir-30a,hsa-mir-30d,5p

hsa-mir-30a,hsa-mir-30e,5p

hsa-mir-30d,hsa-mir-30e,5p

hsa-mir-30b,hsa-mir-30c-1,hsa-mir-30c-2,5p

hsa-mir-323,3p

hsa-mir-323,5p

hsa-mir-328,3p

hsa-mir-329-1,hsa-mir-329-2,3p

hsa-mir-330,3p

hsa-mir-330,5p

hsa-mir-331,3p

hsa-mir-331,5p

hsa-mir-337,3p

hsa-mir-338,5p

hsa-mir-33a,hsa-mir-33b,5p

hsa-mir-342,5p

hsa-mir-34b,3p

hsa-mir-34c,3p

hsa-mir-34c,5p

hsa-mir-361,3p

hsa-mir-362,3p

hsa-mir-362,5p

hsa-mir-582,3p

hsa-mir-582,5p

hsa-mir-369,3p

hsa-mir-369,5p

hsa-mir-370,3p

hsa-mir-371,3p

hsa-mir-374a,hsa-mir-374b,5p

hsa-mir-376b,3p

hsa-mir-378,hsa-mir-422a,5p(hsa-mir-422a),3p(hsa-mir-378)

hsa-mir-380,3p

hsa-mir-382,5p

hsa-mir-383,5p

hsa-mir-409,3p

hsa-mir-409,5p

hsa-mir-429,3p

hsa-mir-432,5p

hsa-mir-433,3p

hsa-mir-449a,5p

hsa-mir-449b,5p

hsa-mir-450a-1,hsa-mir-450a-2,5p

hsa-mir-450b,3p

hsa-mir-450b,5p

hsa-mir-452,5p

hsa-mir-453,3p

hsa-mir-455,5p

hsa-mir-892a,3p

hsa-mir-484,3p

hsa-mir-485,3p

hsa-mir-485,5p

hsa-mir-486__os,3p

hsa-mir-486__os,5p

hsa-mir-487a,3p

hsa-mir-487b,3p

hsa-mir-489,3p

hsa-mir-491,3p

hsa-mir-491,5p

hsa-mir-493,3p

hsa-mir-494,3p

hsa-mir-495,3p

hsa-mir-496,3p

hsa-mir-498,5p

hsa-mir-499,3p

hsa-mir-499,5p

hsa-mir-500,hsa-mir-502,3p

hsa-mir-500,5p

hsa-mir-502,3p

hsa-mir-501,3p

hsa-mir-501,5p

hsa-mir-504,5p

hsa-mir-507,3p

hsa-mir-508,3p

hsa-mir-508,5p

hsa-mir-509-1,hsa-mir-509-2,hsa-mir-509-3,3p

hsa-mir-509-1,hsa-mir-509-3,5p

hsa-mir-509-2,5p

hsa-mir-510,5p

hsa-mir-512-1,hsa-mir-512-2,3p

hsa-mir-513-1,hsa-mir-513-2,3p

hsa-mir-513-1,hsa-mir-513-2,5p

hsa-mir-514-1,hsa-mir-514-2,hsa-mir-514-3,3p

hsa-mir-515-1,hsa-mir-515-2,3p

hsa-mir-516b-1,hsa-mir-516b-2,5p

hsa-mir-516b-1,5p

hsa-mir-517c,hsa-mir-519a-1,hsa-mir-519a-2,3p

hsa-mir-518e,hsa-mir-519a-1,hsa-mir-519b,hsa-mir-519c,hsa-mir-522,hsa-mir-523,5p

hsa-mir-519a-1,hsa-mir-519a-2,3p

hsa-mir-519b,3p

hsa-mir-522,3p

hsa-mir-523,3p

hsa-mir-518a-1,hsa-mir-518a-2,3p

hsa-mir-518a-1,hsa-mir-518a-2,hsa-mir-527,5p

hsa-mir-518a-2,5p

hsa-mir-518d,hsa-mir-518f,hsa-mir-520c-1,hsa-mir-526a-1,hsa-mir-526a-2,5p

hsa-mir-518d,hsa-mir-520c-1,hsa-mir-526a-1,hsa-mir-526a-2,hsa-mir-526b,5p

hsa-mir-520c-1,hsa-mir-520c-2,3p

hsa-mir-519e,3p

hsa-mir-520a,5p

hsa-mir-520d,3p

hsa-mir-520d,5p

hsa-mir-520g,3p

hsa-mir-525,5p

hsa-mir-539,5p

hsa-mir-541,3p

hsa-mir-542,3p

hsa-mir-542,5p

hsa-mir-544,3p

hsa-mir-548a-1,hsa-mir-548a-2,hsa-mir-548a-3,3p

hsa-mir-548a-3,5p

hsa-mir-548b,3p

hsa-mir-548b,5p

hsa-mir-548c,5p

hsa-mir-548d-1,hsa-mir-548d-2,3p

hsa-mir-548d-1,hsa-mir-548d-2,5p

hsa-mir-548d-1,5p

hsa-mir-550-1,hsa-mir-550-2,5p

hsa-mir-556,3p

hsa-mir-556,5p

hsa-mir-561,3p

hsa-mir-576,3p

hsa-mir-576,5p

hsa-mir-579,3p

hsa-mir-584,5p

hsa-mir-589,5p

hsa-mir-593,3p

hsa-mir-597,5p

hsa-mir-598,3p

hsa-mir-616,3p

hsa-mir-618,5p

hsa-mir-624,3p

hsa-mir-627,5p

hsa-mir-628,3p

hsa-mir-628,5p

hsa-mir-629,5p

hsa-mir-636,3p

hsa-mir-642,5p

hsa-mir-651,5p

hsa-mir-653,5p

hsa-mir-7-1,5p

hsa-mir-7-2,5p

hsa-mir-7-3,5p

hsa-mir-9-1,hsa-mir-9-2,5p

hsa-mir-95,3p

ebv-mir-BART18,3p

ebv-mir-BART18,5p

ebv-mir-BART2,3p

ebv-mir-BART20,3p

ebv-mir-BART20,5p

ebv-mir-BART5,rlcv-mir-rL1-8,5p

hcmv-mir-UL148D,3p

hcmv-mir-US25-2,3p

hcmv-mir-US33,3p

hcmv-mir-US33,5p

hcmv-mir-US5-1,3p

kshv-mir-K12-10a,kshv-mir-K12-10b,3p

kshv-mir-K12-11,3p

kshv-mir-K12-5,3p

kshv-mir-K12-6,3p

kshv-mir-K12-8,3p

mghv-mir-M1-1,3p

mghv-mir-M1-5,5p

mghv-mir-M1-7,3p

SI dataset 6. The HMV types in Set I and Set II.

Set I

H3K27ac

H2BK120ac

H3K18ac

H4K5ac

H3K4ac

H3K9me2

H3K14ac

H4K20me3

H3R2me2

H3K27me3

H3K27me2

H4R3me2

H3R2me1

H3K9me3

H3K36me1

H3K79me3

H3K36ac

H4K20me1

H3K9me1

H2BK5me1

H3K79me1

H2BK12ac

H3K4me1

H3K79me2

H3K36me3

Set II

H3K4me2

H2AK5ac

H4K12ac

H3K4me3

H2BK5ac

H3K23ac

H3K9ac

H2AK9ac

CTCF

H3K27me1

H4K16ac

H4K8ac

H4K91ac

H2AZ

H2BK20ac
